# Comparison of thrombus, gut, and oral microbiomes in Korean patients with ST-elevation myocardial infarction: a case–control study

**DOI:** 10.1038/s12276-020-00543-1

**Published:** 2020-12-18

**Authors:** Ju-Seung Kwun, Si-Hyuck Kang, Hyo-Jung Lee, Han-Ki Park, Won-Jae Lee, Chang-Hwan Yoon, Jung-Won Suh, Young-Seok Cho, Tae-Jin Youn, In-Ho Chae

**Affiliations:** 1 Cardiovascular Center, Seoul National University Bundang Hospital, Seongnam-si, Korea; 2 Department of Internal Medicine, Seoul National University College of Medicine, Seoul, Korea; 3 Department of Periodontology, Section of Dentistry, Seoul National University Bundang Hospital, Seongnam-si, Korea; 4 Department of Internal Medicine, Kyungpook National University School of Medicine, Daegu, Korea

**Keywords:** Genetics research, Myocardial infarction

## Abstract

ST-segment elevation myocardial infarction (STEMI) is characterized by thrombotic coronary artery occlusions caused by atherosclerotic plaque rupture. The gut microbiome potentially contributes to the pathogenesis of coronary artery diseases. This study investigated the microbial diversity and composition of coronary thrombi in STEMI patients and the composition of the thrombus microbiome relative to that of the oral and gut microbiomes. A case–control study was performed with 22 STEMI patients and 20 age- and sex-matched healthy controls. Coronary thrombi were acquired from STEMI patients via manual thrombus aspiration during primary coronary intervention. Oral swab and stool samples were collected from both groups, and 16S rRNA sequencing and metagenomic microbiome analyses were performed. Microbial DNA was detected in 4 of 22 coronary thrombi. *Proteobacteria* (*p*) and *Bacteroidetes* (*p*) were the most abundant phyla. The oral and gut microbiomes significantly differed between patients and healthy controls. The patient group presented microbial dysbiosis, as follows: a higher relative abundance of *Proteobacteria* (*p*) and *Enterobacteriaceae* (*f*) in the gut microbiome and a lower abundance of *Firmicutes* (*p*) and *Haemophilus* (*g*) in the oral microbiome. Furthermore, 4 significantly abundant genera were observed in the coronary thrombus in the patients: *Escherichia*, 1.25%; *Parabacteroides*, 0.25%*; Christensenella*, 0.0%; and *Bacteroides*, 7.48%. The present results indicate that the relative abundance of the gut and oral microbiomes was correlated with that of the thrombus microbiome.

## Heart disease: microbiome linked to heart attack

Disruption to microbiome composition and functioning may contribute to heart attacks. The most serious form of heart attack is ST-segment elevation myocardial infarction (STEMI), where the coronary artery is blocked by ruptured plaques. Previous research has linked cardiovascular diseases with disruption to the microbiome, but links between STEMI and the microbiome are not yet clear. Si-Hyuck Kang at Seoul National University in Seongnam-si, South Korea, and co-workers analyzed oral, fecal, and coronary thrombus (blood clot) microbial samples from 22 STEMI patients and 20 healthy controls. They found significant differences in oral and gut microbiome composition between the two groups, including increased *Proteobacteria* phylum and *Enterobacteriaceae* species and decreased *Firmicutes* phylum in STEMI patients. Microbes matching patients’ oral and gut bacteria were present in four thrombus samples, suggesting that microbes may influence clot formation and plaque rupture.

## Introduction

ST-segment elevation myocardial infarction (STEMI) is a catastrophic condition that is associated with high mortality rates and a substantial risk of complications^1^. Atherosclerotic plaques typically progress over years or decades^2^. However, thrombotic cascades of STEMI occur without warning. Plaque rupture has been proposed as a key mechanism underlying coronary thrombosis^3^. Accumulating evidence has linked local and systemic inflammation with plaque rupture. Inflammatory cells are observed at the site of ruptured plaques. Systemic inflammatory reactions reportedly cause plaque inflammation, leading to thinning of the fibrous cap and acute plaque rupture.

The human microbiome significantly contributes to host metabolism^4^. Dysbiosis of the microbial ecosystem is associated with various human diseases, including cardiovascular diseases^5^. The intestinal microbiota is associated with major cardiovascular risk factors, such as hypertension, diabetes, dyslipidemia, and obesity^6–8^. Previous studies have reported the role of intestinal microbiota-derived physiological modulators (e.g., short-chain fatty acids) and pathogenic mediators (e.g., trimethylamine N-oxide) in host susceptibility to cardiovascular diseases. However, details regarding the association between microbiomes and acute thrombotic conditions, such as STEMI, remain elusive.

Previous studies have reported the presence of bacterial signatures in coronary thrombi^9–11^. Other studies have reported that gut microbe-derived proatherosclerotic metabolites, such as trimethylamine-N-oxide, are potentially associated with cardiovascular disease^12,13^. However, no studies have focused on the association between the thrombus microbiome and host microbial ecology in its entirety. Recent advancements in next-generation sequencing have provided detailed insights into the human microbiome with unprecedented resolution^14^. Furthermore, metagenomics has helped identify diverse microbial communities and profile highly complex networks^15^.

This study aimed to investigate (1) the coronary thrombus microbiome and (2) its composition relative to the gut and oral microbiomes of STEMI patients relative to those of healthy individuals.

## Materials and methods

The data supporting the present results are available from the corresponding author upon reasonable request.

### Study design, subjects, and samples

Our case–control study enrolled 22 STEMI patients and 20 age-matched and sex-matched healthy controls. Patients were enrolled at Seoul National University, Bundang Hospital. Inclusion criteria for the patient group were as follows: (i) ECG criteria of at least 1 mm in 2 or more standard leads or at least 2 mm in 2 or more contiguous precordial leads of ST-segment elevation or new left bundle-branch block; and (ii) angiographically proven coronary thrombi. The exclusion criteria for patients were as follows: (i) other identifiable etiologies of coronary thrombi (e.g., coronary vasospasm and systemic thromboembolism); and (ii) evident active infection during admission. Control subjects were enrolled voluntarily through poster advertisements among those undergoing routine health checks at the same institution. Twenty controls with no previous history of cardiovascular disease and no evidence of active infection were matched 1:1 to each patient by age (by 5 years) and sex. All study subjects were ethnic Koreans. Oral swab and stool samples were collected from both groups (AccuBuccal and AccuStool collection kits, AccuGene, Incheon, Korea). This study was approved by the local institutional review board (B-1801-444-301). Informed consent was obtained from all study participants in accordance with the tenets of the Declaration of Helsinki.

### Sample collection, DNA extraction, and pyrosequencing

Coronary thrombi were acquired from STEMI patients using manual thrombus aspiration during primary coronary intervention (Supplementary Fig. 1). Oral swab and stool samples were collected from both groups using dedicated kits (AccuBuccal and AccuStool collection kit, AccuGene). The samples were immediately frozen at −20 °C and stored at −70 °C within 24 h. Bacterial DNA was extracted from these samples.

### DNA extraction and quantification

DNA was extracted using a DNeasy PowerSoil Kit (Qiagen, Hilden, Germany) in accordance with the manufacturer’s instructions. Extracted DNA was quantified using Quant-IT PicoGreen (Invitrogen, CA, USA).

### Library construction and sequencing

The sequencing library was prepared using Illumina 16S Metagenomic Sequencing Library protocols to amplify the V3 and V4 regions. Input gDNA (2 ng) was amplified via PCR with 1× reaction buffer, 1 nM dNTP mix, 500 nM each of the universal F/R PCR primers, and 2.5 U of Herculase II fusion DNA polymerase (Agilent Technologies, Santa Clara, CA, USA) with the following cycling conditions: 3 min at 95 °C for heat activation, followed by 25 cycles of 30 s at 95 °C, 30 s at 55 °C, and 30 s at 72 °C, and a final 5-min extension at 72 °C. The universal primer pair with Illumina adapter overhang sequences used for initial amplification was as follows: V3-F: 5′-TCGTCGGCAGCGTCAGATGTGTATAAGAGACAGCCTACGGGNGGCWGCAG-3′ and V4-R: 5′-GTCTCGTGGGCTCGGAGATGTGTATAAGAGACAGGACTACHVGGGTATCTAATCC-3′. The first PCR product was purified with AMPure beads (Agencourt Bioscience, Beverly, MA, USA). Following purification, 10 µL of the first PCR product was PCR amplified for final library construction containing the index using NexteraXT Indexed Primer. The cycle for the second PCR was the same as that for the first PCR, except for using 10 cycles. The PCR product was purified using AMPure beads. The final purified product was then quantified using qPCR in accordance with the qPCR Quantification Protocol Guide (KAPA Library Quantification kits for Illumina Sequencing Platforms) and qualified using a TapeStation D1000 ScreenTape (Agilent Technologies, Waldbronn, Germany). Paired-end (2 × 300 bp) sequencing was performed by Macrogen (Seoul, Korea) using the MiSeq™ platform (Illumina, San Diego, CA, USA)

### Preprocessing, clustering, and taxonomic assignment

Raw paired-end 16S rDNA reads (V3-V4 region) were merged into consensus fragments via FLASH (version 1.2.11)^16^. Filtered reads were clustered at 97% identity using CD-HIT-OTU^17^ by identifying chimeric reads and eliminating low-quality, ambiguous, and short (<400 bp) reads. The remaining representative reads were clustered using a greedy algorithm into operational taxonomic units (OTUs).

Taxonomic assignment was conducted via BLAST (version 2.4.0)^18^. Microbial community analyses, such as alpha-diversity and beta-diversity analyses and principal coordinate analyses, were performed using quantitative insights into microbial ecology (QIIME v1.0) software^19^. Alpha-diversity was evaluated using the Chao1, Shannon and inverse Simpson indices. Beta-diversity was assessed via principal coordinate analysis (PCoA) based on weighted UniFrac distance metrics. Normality was tested using Shapiro-Wilk’s test, and differences in relative abundance were assessed using Student’s *t*-test or Wilcoxon rank sum test, as appropriate. The false discovery rate (FDR) was used to correct for multiple hypothesis testing^20^. Linear discriminant analysis (LDA) effect size (LEfSe) was used to discover metagenomic biomarkers through class comparison, tests for biological consistency, and effect size estimation^21^. Heatmap and PCoA analyses were used to visualize differentially abundant functional categories (LDA score ≥2.0). Analysis of similarities (ANOSIM) was performed to compare the matrix of rank dissimilarity. Functional gene enrichment analysis was performed through phylogenetic investigation of communities by reconstructing unobserved states (PICRUSt), which provides proportional contributions of KEGG categories of each sample^22,23^.

## Results

### Microbial diversity

Coronary thrombi were harvested from 22 STEMI patients. Their mean age was 59.4 years, and 91% were male. While oral swab samples were collected from all patients, only 20 patients provided stool samples. Oral swab and stool samples were collected from 20 age-matched and sex-matched controls who were free of cardiovascular disease. The baseline characteristics of the patients are shown in Table 1.

**Table 1:** Profile of the study population.

Characteristics	STEMI (*N* = 22)	Control (*N* = 20)
Age (in years)	59.4 ± 11.4	59.7 ± 11.8
Male sex	20 (90.9%)	18 (90.0%)
Hypertension	13 (59.1%)	
Diabetes	6 (27.3%)	
Dyslipidemia	3 (13.6%)	
Smoking		
Current smoker	10 (45.4%)	
Former smoker	6 (27.3%)	
Never smoker	6 (27.3%)	
History of stroke	0 (0.0%)	
Family history of premature coronary disease	4 (18.2%)	
Killip class		
I	19 (86.4%)	
II	0 (0.0%)	
III	1 (4.5%)	
IV	2 (0.9%)	
Coronary disease extent		
1-vessel disease	10 (45.5%)	
2-vessel disease	7 (31.8%)	
3-vessel disease	5 (13.6%)	

^*^STEMI denotes ST-elevation myocardial infarction

**Fig. 1:**
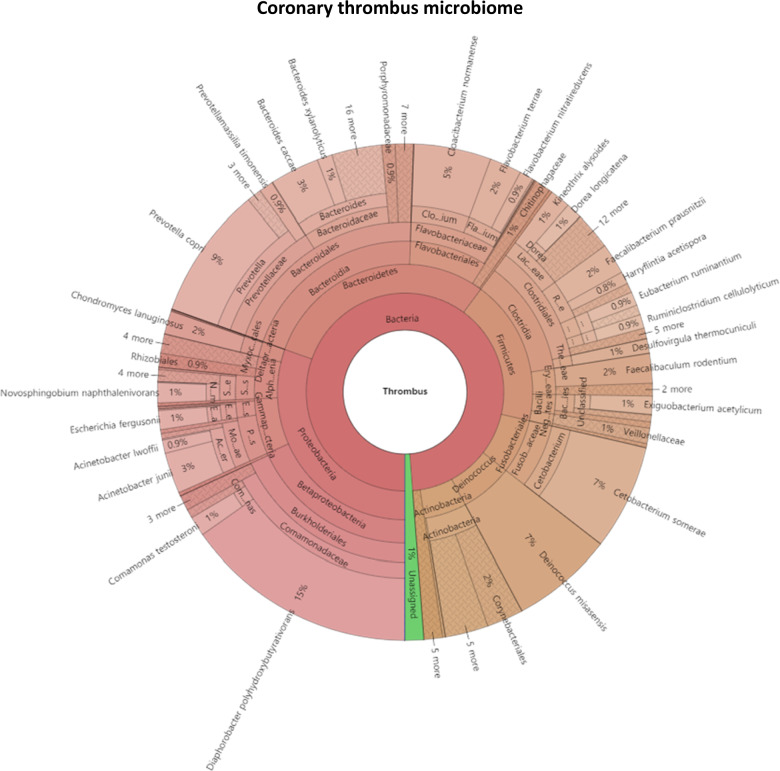
Coronary thrombus microbiome. Krona chart of the bacteria represented by 16S rRNA sequences recovered from coronary thrombi.

16S rRNA genes were detected in 4 of 22 coronary thrombi. The remaining 18 samples did not pass quality control analysis owing to multiple peaks or inadequate DNA. The microbial composition of the thrombi varied widely across individuals. *Proteobacteria* (*p*) and *Bacteroidetes* (*p*) were the most abundant phyla among the detected microbes (Fig. 1). We found 244 species from 188 genera, 100 families, 50 orders, 26 classes, and 11 phyla among the thrombus samples. Supplementary Fig. 2 shows the bacterial composition of the oral and stool samples of the patient and control groups.

First, microbial diversity was measured. The alpha-diversity of the stool microbiome, defined as the number of species present within each sample, did not differ significantly between groups (stool sample: *P* = 0.820 by the Shannon index, *P* = 0.799 by the inverse Simpson index; oral sample: *P* = 0.075 by the Shannon index, *P* = 0.299 by the inverse Simpson index) (Supplementary Fig. 3). However, the oral microbiome of STEMI patients displayed significantly higher alpha-diversity than that of the control subjects (*P* = 0.032, *P* = 0.032, *P* = 0.075, and *P* = 0.299 for each index).

### Differences in microbial composition between STEMI patients and healthy controls

**Fig. 2:**
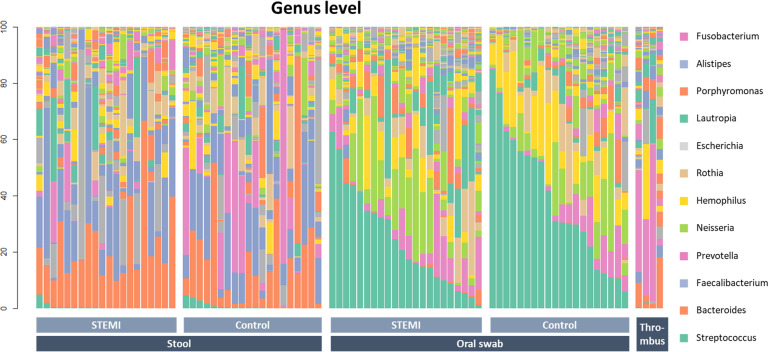
Relative abundance of microbiome at the genus level. Bar plot showing relative abundance at the genus level in the stool, oral, and thrombus microbiomes of STEMI patients and control groups.

**Fig. 3:**
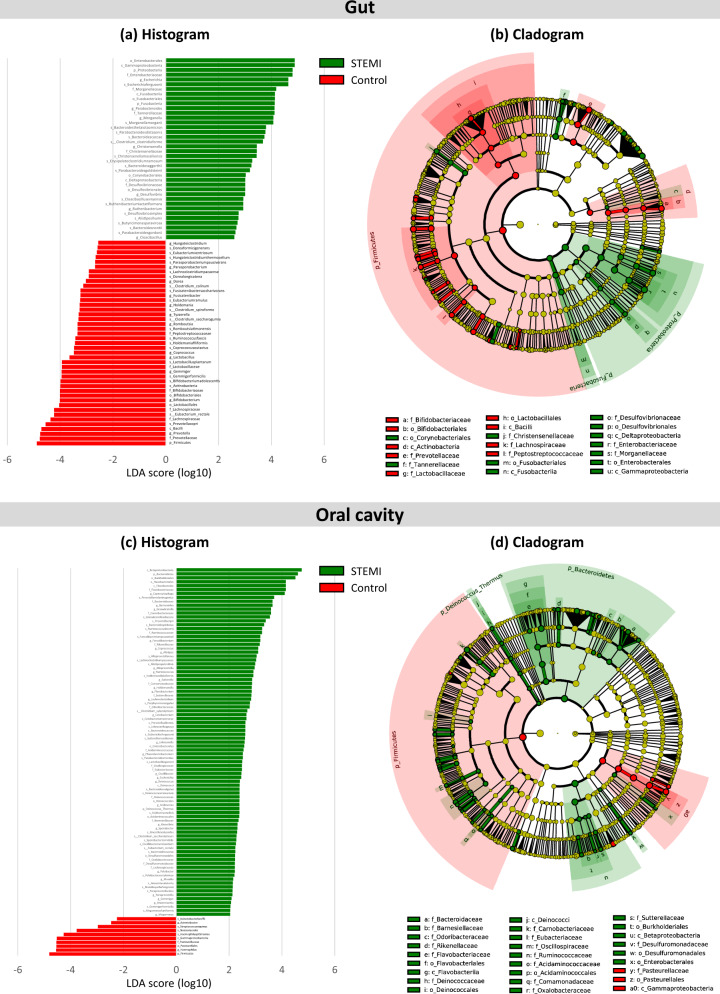
Differentially abundant taxa. Linear discriminant analysis (LDA) effect size (LEfSe) was performed on the microbial communities of the stool and oral swab samples. **a** Histogram of the LDA scores for differentially abundant bacterial taxa in the stool sample between the patient and control groups. **b** Taxonomic cladogram of the gut microbiota. **c** Histogram and **d** cladogram of the oral microbiota. Green and red colors represent bacterial taxa that were significantly overrepresented in the STEMI patients and control groups, respectively.

The oral and gut microbiomes of the patient and control groups significantly differed. Figure 2 displays the general landscape of the microbial composition of all samples at the genus level. Furthermore, taxonomic assignments at the other levels (phylum, class, order, family, and species) are also shown in Supplementary Fig. 4. Supplementary Fig. 5 displays a comparison among the oral, stool, and thrombus microbiota side-by-side among the 4 patients among whom the thrombus microbiome was detected. High-dimensional class comparisons were performed for taxa displaying a significantly different abundance between the STEMI and control groups (Fig. 3). LEfSe screened 81 clades from the gut microbiome. The patient group displayed dysbiosis of the gut microbiota. The prevalence of *Proteobacteria* (*p*) and *Enterobacteriaceae* (*f*) was significantly higher in the patients than in the controls (18.8% vs. 6.1% [*P* = 0.010]; and 14.0% vs. 2.0% [*P* = 0.001]), while that of *Firmicutes* (*p*) and *Lactobacillales* (*o*) was significantly lower (40.9% vs. 56.4% [*P* = 0.006]; and 1.7% vs. 3.5% [*P* = 0.008])^24^. Short-chain fatty acid (SCFA)-producing bacteria displayed a lower abundance (*Prevotella* (*g*)*,* 0.38% vs. 11.4% [*P* = 0.004]; *Lachnospiraceae* (*f*), 4.2% vs. 5.0%; [*P* = 0.013]); *Eubacterium rectale* (*s*)*,* 0.84% vs. 4.66 [*P* = 0.003]; *Lactobacillales* (*o*), 1.7% vs. 3.5% [*P* = 0.009]; and *Bifidobacterium* (*g*) 0.8% vs. 2.4% [*P* = 0.024])^25^.

Based on a LDA threshold score of 2.0, a total of 99 clades were screened from the oral sample. The relative abundance of *Porphyromonas gingivalis(s)* did not differ significantly (2.9% vs. 1.8% [*P* = 0.695]). The oral microbiota in the patient group also showed dysbiosis. *Firmicutes* (*p*) (34.9% vs. 48.2% [*P* = 0.031]) and *Haemophilus* (*g*) (5.8% vs. 12.4% [*P* = 0.028]) were significantly depleted, while lipopolysaccharide (LPS)-producing bacteria displayed a higher prevalence (*Bacteroidetes* (*p*), 19.6% vs. 11.0% [*P* = 0.010], and *Bacteroides* (*g*) (0.86% vs. 0.05% [*P* < 0.001]).

**Fig. 4:**
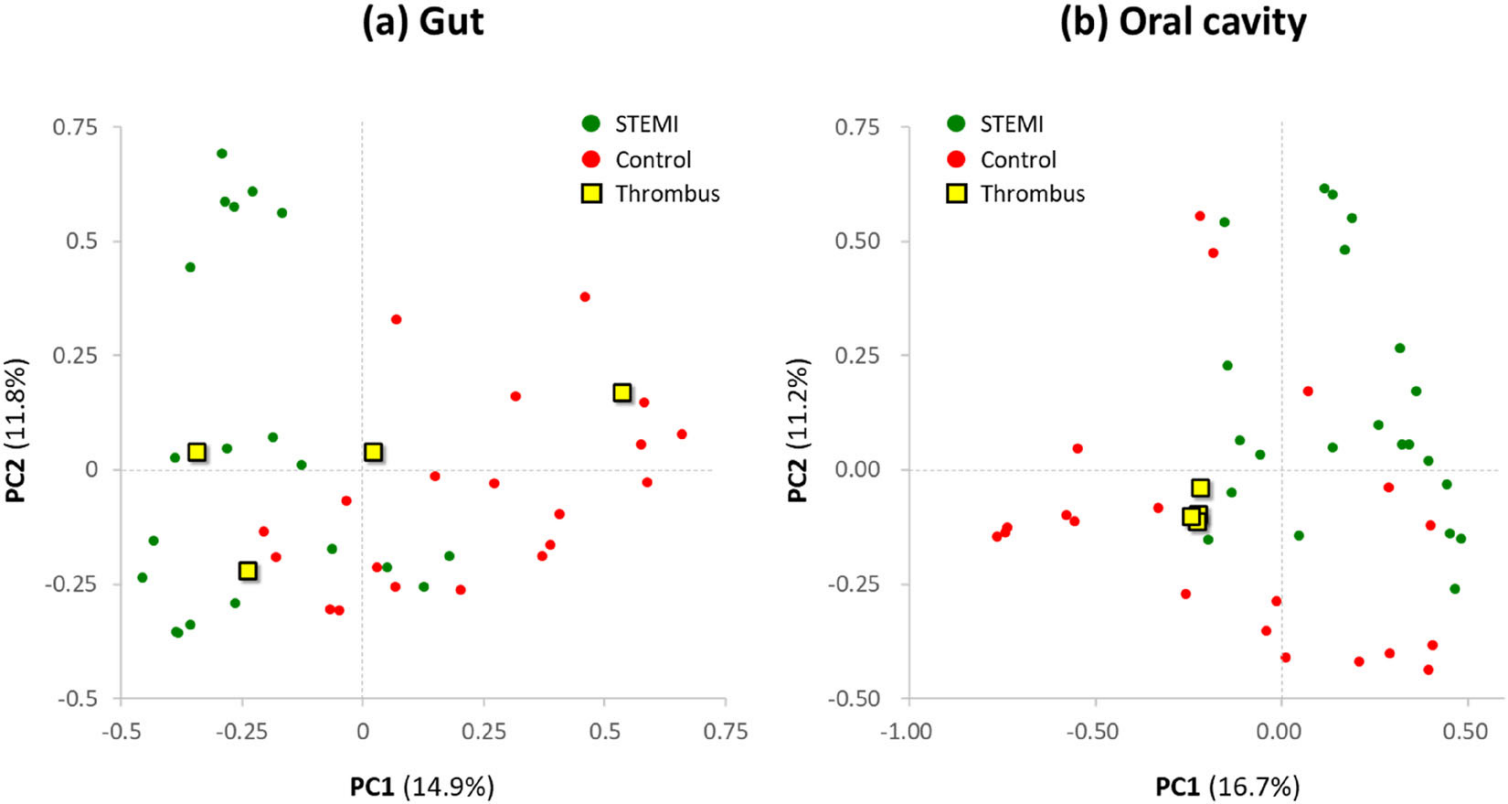
Beta-diversity of the thrombus microbiome using principal coordinate analysis. **a** the gut microbiome and **b** the oral microbiome.

We assessed the relationships among bacterial communities from their beta-diversity to generate PCoA plots. OTUs of the thrombus microbiome relative to those of the gut and oral microbiomes displayed different clustering between groups (Fig. 4), suggesting phylogenetic closeness between microbial communities within each group. Supplementary Fig. 6 displays the OTUs of both the stool and oral microbiomes. Furthermore, PCoA for the patients with the thrombus microbiome and the matched controls is indicated in Supplementary Fig. 7.

Differences in the relative abundance of taxa between the patient and control groups were compared. *Escherichia* (*g*) (10.3% vs. 1.46%, false discovery rate [FDR] adjusted *P*-value = 0.029), *Parabacteroides* (*g*) (3.23% vs. 0.62%, FDR-adjusted *P* = 0.029), and *Christensenella* (*g*) (0.67% vs. 0.17%, FDR-adjusted *P* = 0.029) were significantly enriched among the gut microbiome constituents in the patient group, while *Lactobacillus* (*g*) was significantly less abundant (1.12% vs. 2.54%, FDR-adjusted *P* = 0.029) (Supplementary Table 1). *Bacteroides (g)* displayed a significantly higher abundance among the oral microbiome in the patient group (0.86% vs. 0.03%, FDR-adjusted *P* = 0.007) (Supplementary Table 2). The relative abundances of *Escherichia* (*g*)*, Parabacteroides* (*g*)*, Christensenella* (*g*), and *Bacteroides* (*g*) in the coronary thrombus microbiome were 1.25%, 0.25%, 0.0%, and 7.48%, respectively.

**Fig. 5:**
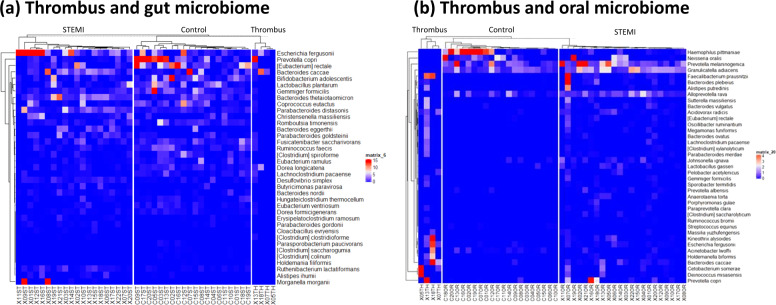
Heatmap of the selected most differentially abundant features at the species level. Relative abundance ratios of selected biomarkers are depicted for: **a** stool and thrombus and **b** oral swab and thrombus. Blue represents lower abundance, white represents intermediate abundance, and red represents the highest abundance.

The thrombus microbiome was assessed relative to the gut and oral microbiomes via heatmaps based on OTU abundance (Fig. 5). Thirty-seven representative features were selected at the species level based on the LEfSe of the gut microbiome, and 39 species were selected from the oral microbiome. The relative abundance of the thrombus microbiome was also plotted via the heatmap. Taxonomic communities displayed differential abundance between the patient and control groups at each site. Figure 5b visually shows that the thrombus microbiome exhibited a taxonomic distribution that was similar to that of the oral microbiome of STEMI patients (Fig. 5b). Supplementary Fig. 8 displays a side-by-side comparison of the relative abundance of the 4 individual patients with thrombus samples. ANOSIM corroborated that the dissimilarity between the stool and thrombus samples was larger than that between the oral and thrombus samples (unweighted *R* = 0.444 and 0.2916, respectively) (Supplementary Fig. 9).

### Different metabolic pathways of microbial communities between STEMI and control subjects

**Fig. 6:**
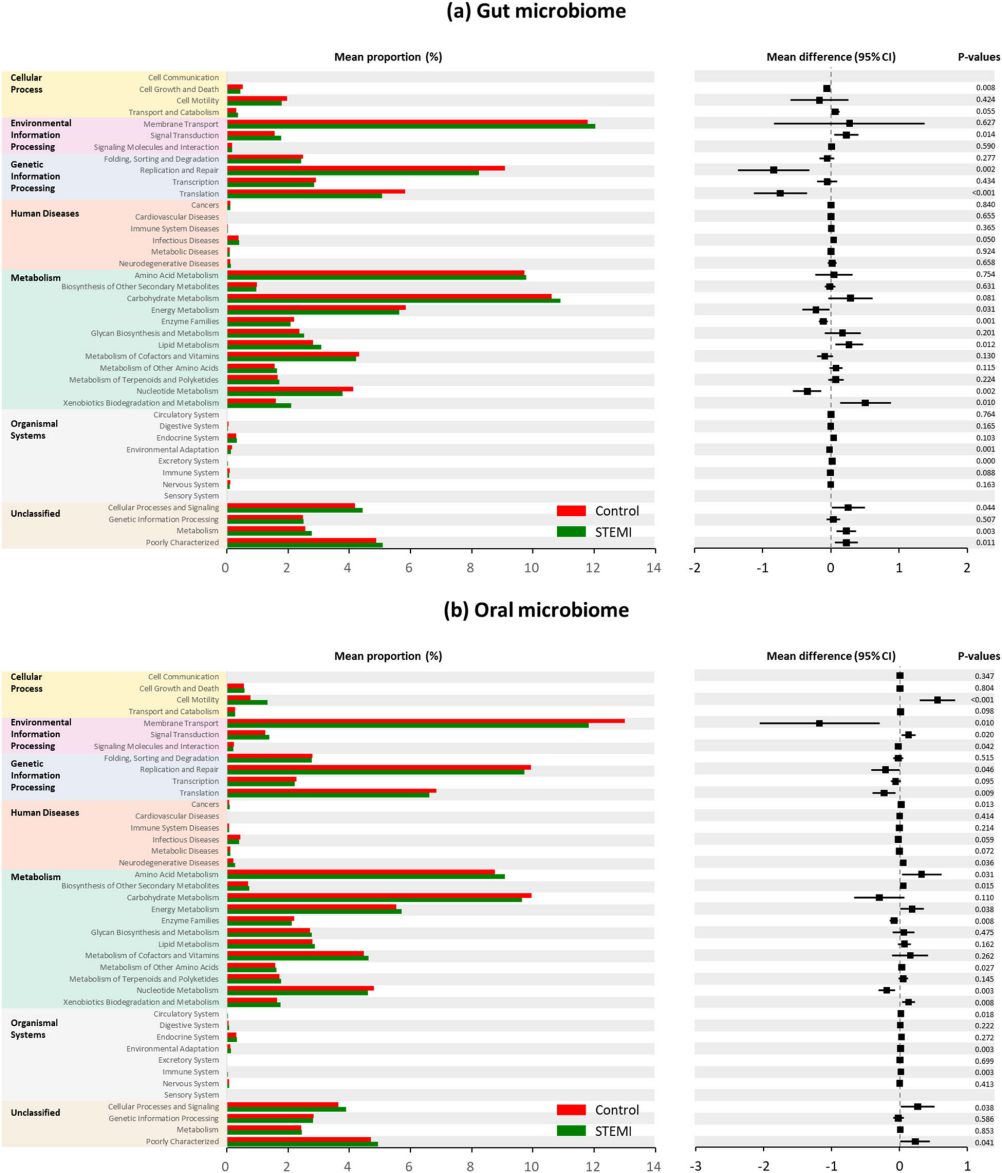
Metabolic implication. Different metabolic pathways of microbial communities between the: **a** gut microbiome and **b** oral microbiome groups using the PICRUSt algorithm. The STEMI patients and control groups exhibited significant differences among the 41 KEGG pathways.

Bacterial gene functions were predicted via 16S rRNA gene-based microbial compositions using the PICRUSt algorithm to make inferences from KEGG annotated databases. The STEMI and control groups exhibited significant differences among the 41 KEGG pathways (Fig. 6). The stool microbiome displayed differences in cell growth and death, signal transduction, replication and repair, translation, infectious diseases, energy metabolism, enzyme families, lipid metabolism, nucleotide metabolism, xenobiotic biodegradation and metabolism, cellular processes and signaling, and metabolism. The oral microbiome displayed differences in cell motility, membrane transport, signal transduction, signaling molecules and interaction, replication and repair, translation, cancer, neurodegenerative diseases, amino acid metabolism, biosynthesis of other secondary metabolites, energy metabolism, enzyme families, metabolism of other amino acids, nucleotide metabolism, circulatory system, environmental adaptation, immune system, and cellular processes and signaling.

## Discussion

Numerous diverse microbial species reside in various tissues of the human body, including the skin, oral mucosa, and gastrointestinal tract. Imbalances in the microbiota are associated with various human diseases that affect regions beyond the local anatomic site, such as neurologic and cardiovascular disorders^4^. Efforts have been made to define microbial components of the human genetic and metabolic landscape in order to better understand the underlying pathophysiology and develop novel therapeutic strategies^26^.

The current study comprehensively analyzed the thrombus, gut, and oral microbiomes of STEMI patients relative to those of age-matched and sex-matched healthy controls. The study indicated substantial differences in the composition and function of the stool and oral microbiomes between the two groups. Furthermore, 4 of 22 coronary thrombi acquired from STEMI patients were positive for bacteria. Microbes that were significantly abundant in the oral and stool microbiomes were also significantly abundant in that of the coronary thrombus.

Previous studies have reported an association between various infectious agents and human atherosclerotic diseases. Previous studies have reported the pathogenic role of *Chlamydia pneumoniae* and cytomegalovirus in atherosclerotic progression^27–29^. Such reports have led to interventional studies based on the hypothesis that antibiotic therapy may help prevent atherosclerosis. However, randomized trials using macrolide antibiotic therapies have failed to provide benefits in preventing future cardiovascular events^30–33^.

The presence of microbial DNA in coronary thrombi has been demonstrated previously. Studies using targeted sequencing via PCR have detected periodontal bacteria, such as *Viridans streptococci*^9,10^. Hansen et al. performed next-generation sequencing, which demonstrated a relatively high abundance of *Pseudomonas aeruginosa* DNA. Furthermore, they confirmed the presence of whole and intact bacteria as biofilm microcolonies in selected thrombi^11^. Advancements in next-generation sequencing and metagenomic analysis have facilitated the analysis of microbiomes in carotid and coronary atherosclerotic plaques^34–36^. Koren et al. reported that bacteria present in atherosclerotic plaques may have originated in the oral cavity and gut microbiota^37^.

The present results indicate that microbial dysbiosis may be the mechanism underlying acute myocardial infarction. This study reports an increased abundance of *Proteobacteria* (*p*) and *Enterobacteriaceae* (*f*) among STEMI patients. The phylum *Proteobacteria* normally constitutes a minor proportion of the natural human gut flora. Recent studies have suggested that an increased prevalence of *Proteobacteria*, frequently observed in metabolic disorders, represents gut dysbiosis^24^. Jie et al. reported that the abundance of *Enterobacteriaceae* (*f*) among patients with atherosclerotic cardiovascular disease is higher than that among healthy controls^38^. A lower abundance of SCFA-producing bacteria and a higher abundance of LPS-producing bacteria were concurrent with previous reports^5,39,40^.

This study comprehensively analyzed the thrombus, gut, and oral microbiomes of STEMI patients. Koren et al. performed a metagenomic analysis to survey the bacterial diversity of atherosclerotic plaques and oral and gut samples of patients undergoing carotid endarterectomy^37^. The present results differ from those of Koren since we enrolled STEMI patients at an active phase and acquired coronary thrombus. STEMI results from plaque rupture, which marks a very specific event of chronic atherosclerotic changes lasting years or decades. The difference in the study designs led to a significant difference in study findings. While Koren et al. reported that the patients’ microbiota was similar to that of control subjects, this study reports marked differences in the microbiota between the patient and control groups. We assume that these differences in the results were observed because we enrolled patients with acute-stage STEMI. Technically, more extensive sequencing with higher resolution was performed in this study.

One major study limitation was that microbial DNA was detected only in 4 of 22 thrombus samples. However, this fact does not indicate that microbial DNA was not present in the remaining 18 samples. The samples did not pass quality control analysis after library construction owing to multiple peaks and low concentrations. Thrombi used herein were usually small fragments acquired during primary coronary interventions (Supplementary Fig. 1). These thrombi varied widely in weight and composition, such as platelet-rich and fibrin-rich thrombi, across clinical situations. Such variables were not obtained in this study and should be considered in future studies.

A clinical implication of the current study is that microbes surrounding the human body may play a role in plaque rupture and ultimately contribute to fatal cardiovascular diseases, such as acute myocardial infarction. However, because this was a case–control study, we were only able to assess relationships rather than to infer causal relationships. Stool and oral swab samples were collected during admission for post-myocardial infarction, which limits temporality. Zhou et al. recently reported that intestinal permeability increased following myocardial infarction, leading to higher microbial richness and diversity in the systemic microbiome of STEMI patients, where >12% of post-STEMI blood bacteria were dominated by intestinal microbiota constituents (*Lactobacillus*, *Bacteroides*, and *Streptococcus*)^41^. Furthermore, they linked adverse outcomes of myocardial infarction to the translocation of intestinal microbiota into systemic circulation.

This study demonstrated that microbial communities were present in human coronary thrombi and that these communities were correlated with those in the gut and oral microbiomes. Future studies are needed to further identify the clinical implications of these findings and to apply them to develop novel therapeutic strategies. For example, the role of the gut and tumor microbiomes is being actively investigated by oncologists. Studies have confirmed the presence of bacteria in tumors, suggesting that these bacteria mediate the tumor response to chemotherapy and immunotherapy among cancer patients^42,43^. Other studies have reported that the commensal microbiota has an impact on the future risk of cancer^44,45^.

The current study detected bacterial DNA in the coronary thrombi of STEMI patients. The gut and oral microbiomes of these patients displayed significant differences compared with those of age-matched and sex-matched controls. The relative abundance of the gut and oral microbiomes was correlated with that of the thrombus microbiome. Our findings indicate that commensal microbiota may play a role in plaque rupture and thrombus formation in acute myocardial infarction.

## Supplementary information

Supplementary File
