# Supplementary material for: Comparison of thrombus, gut, and oral microbiomes in Korean patients with ST-elevation myocardial infarction: a case–control study
Source: Exp Mol Med. 2020 Dec 18;52(12):2069–79. doi: 10.1038/s12276-020-00543-1 (PMC8080616; doi:10.1038/s12276-020-00543-1)
Supplement: Supplementary file 1 — Supplementary File [file 12276_2020_543_MOESM1_ESM.pdf]

Supplementary Figure 1. Acquisition of coronary thrombus. (a) Thrombus aspiration using a manual suction device during primary coronary intervention. (b) Acquired thrombus using a dedicated filter. (c) Samples were moved and stored in an Eppendorf tube filled with 0.9% normal saline.

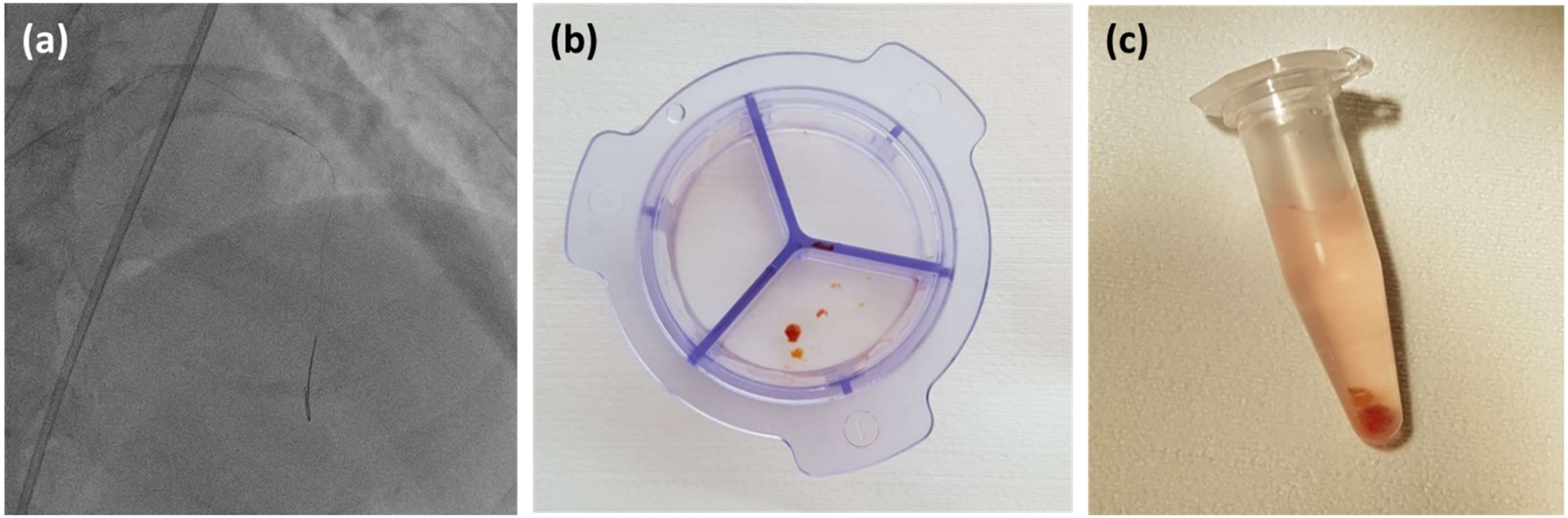

Supplementary Figure 2. Krona chart of the bacteria represented by 16S rRNA sequences recovered (a) the gut microbiome and (b) the oral microbiome.

(a) Gut

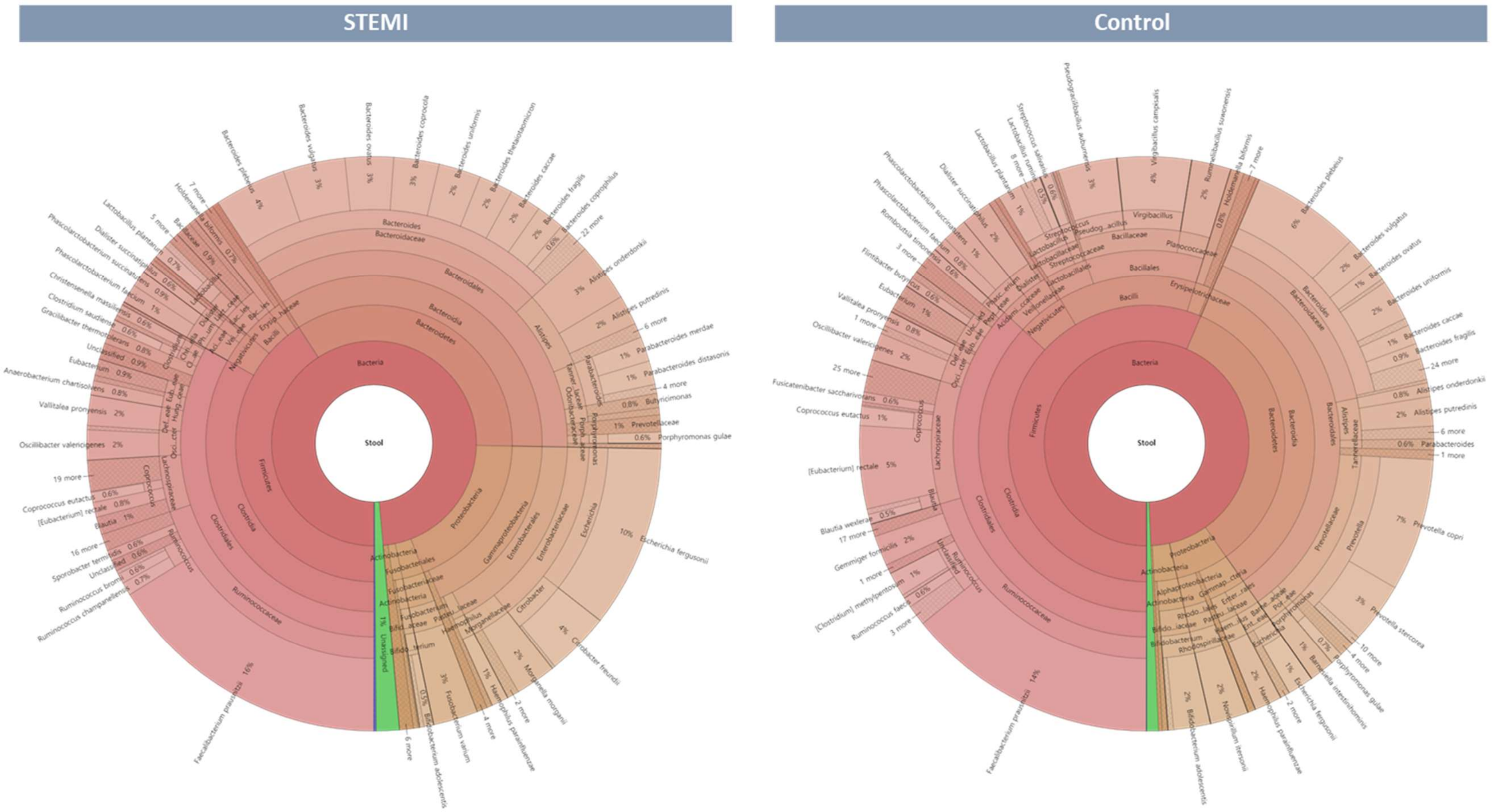

(b) Oral cavity

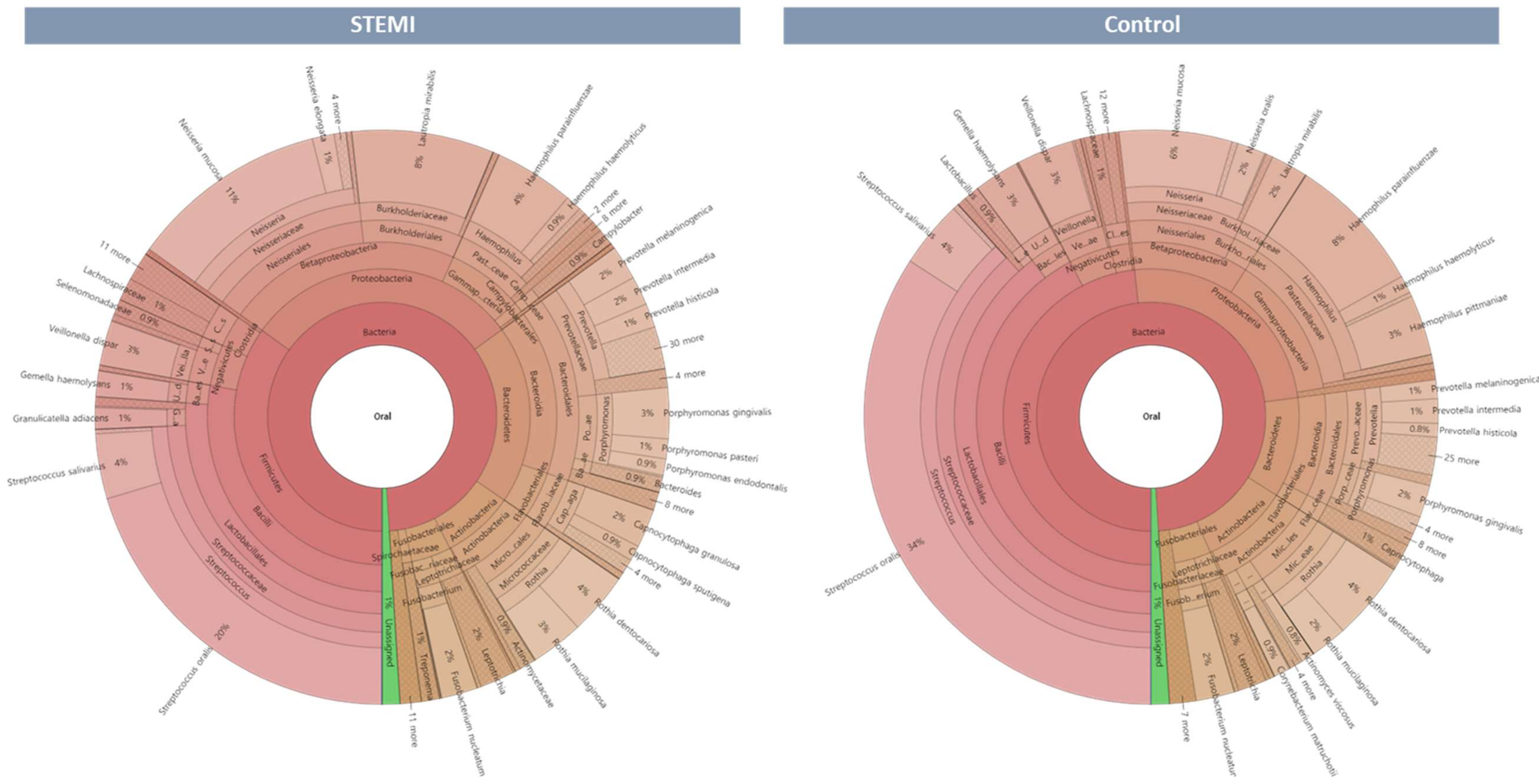

Supplementary Figure 3. Alpha-diversity of (a) the gut microbiome and (b) the oral microbiome did not differ significantly between the groups (Stool sample: P=0.820 by the Shannon index, P=0.799 by the inverse Simpson index; oral sample: P=0.075 by the Shannon index, P=0.299 by the inverse Simpson index).

(a) Gut

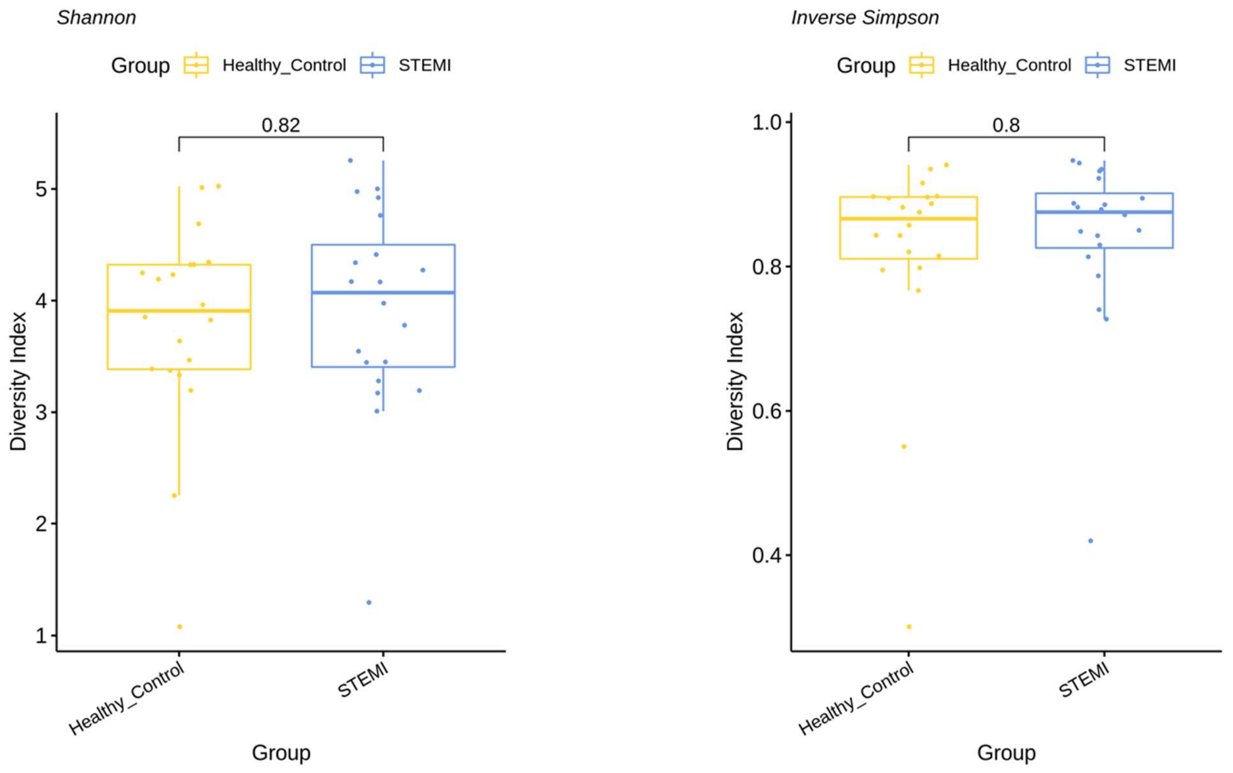

(b) Oral

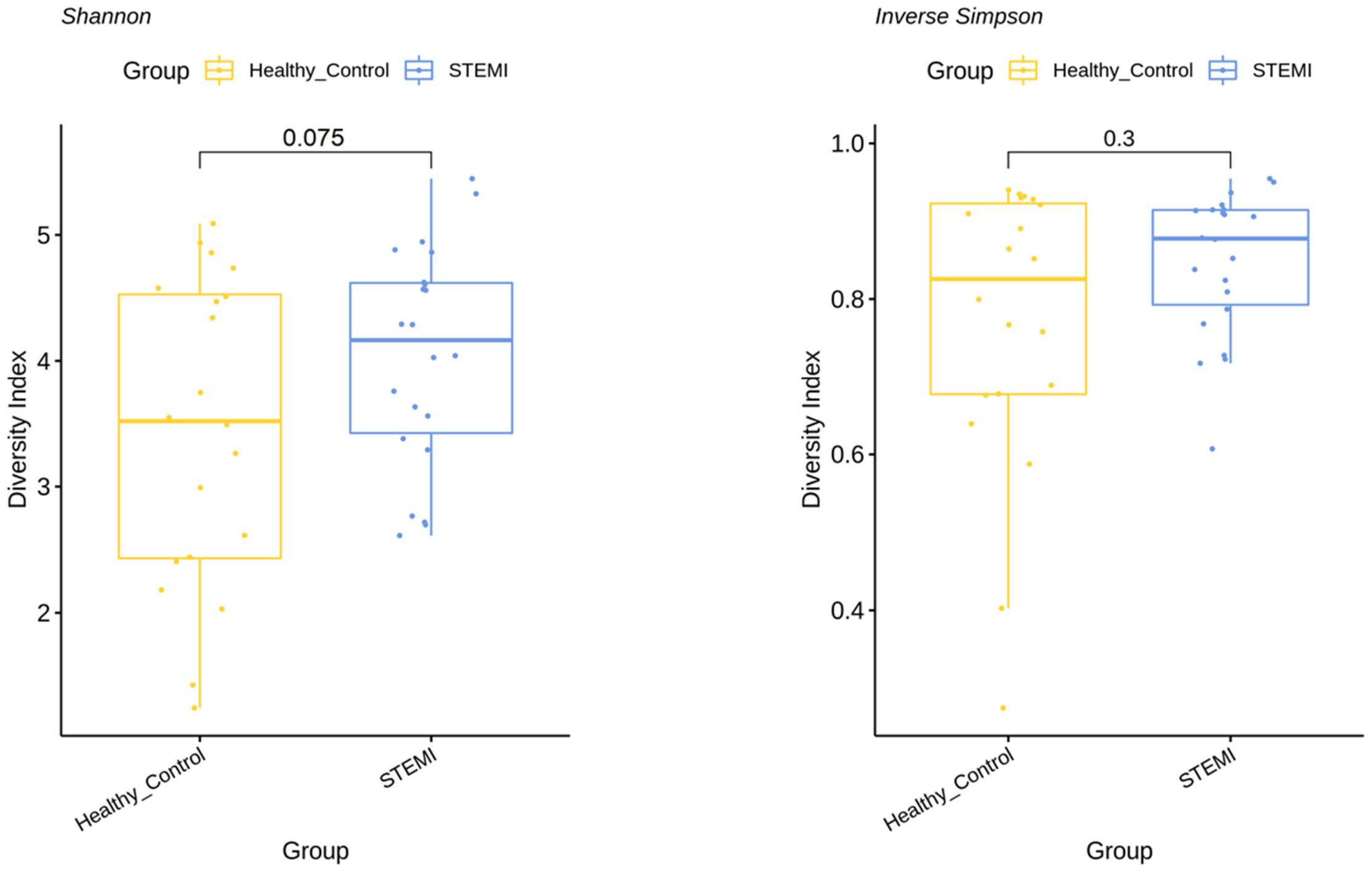
Shannon
Group
Healthy_Control
STEMI
0.075
Diversity Index
5
4
3
2
Healthy_Control
STEMI
Group
Inverse Simpson
Group
Healthy_Control
STEMI
0.3
Diversity Index
1.0
0.8
0.6
0.4
Healthy_Control
STEMI
Group

Supplementary Figure 4. Bar plot showing relative abundance at each taxonomic level ((a) Phylum, (b) Class, (c) Order, (d) Family, (e) Species except for the genus level which is presented in Figure 2) in the stool, oral, and thrombus microbiome of the patient and control groups.

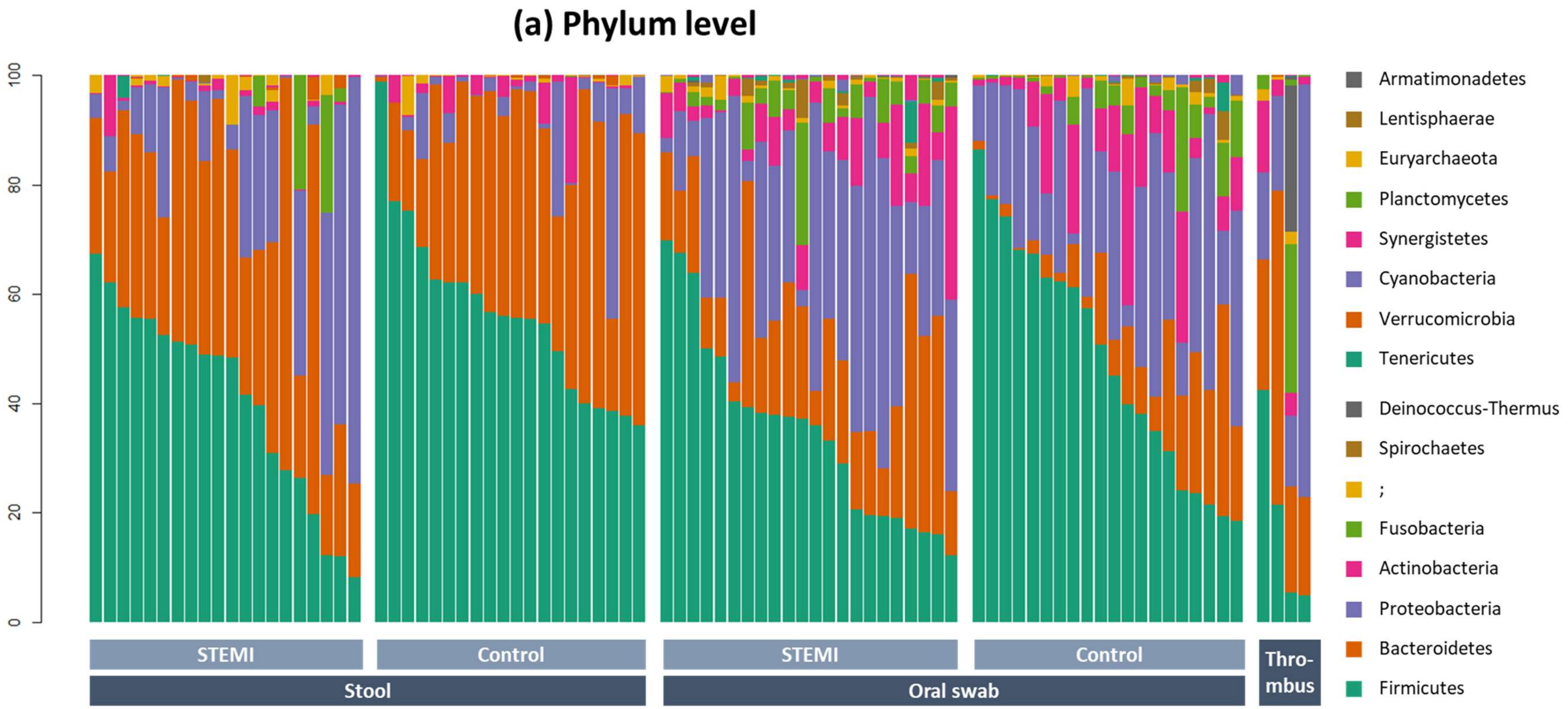

(b) Class level

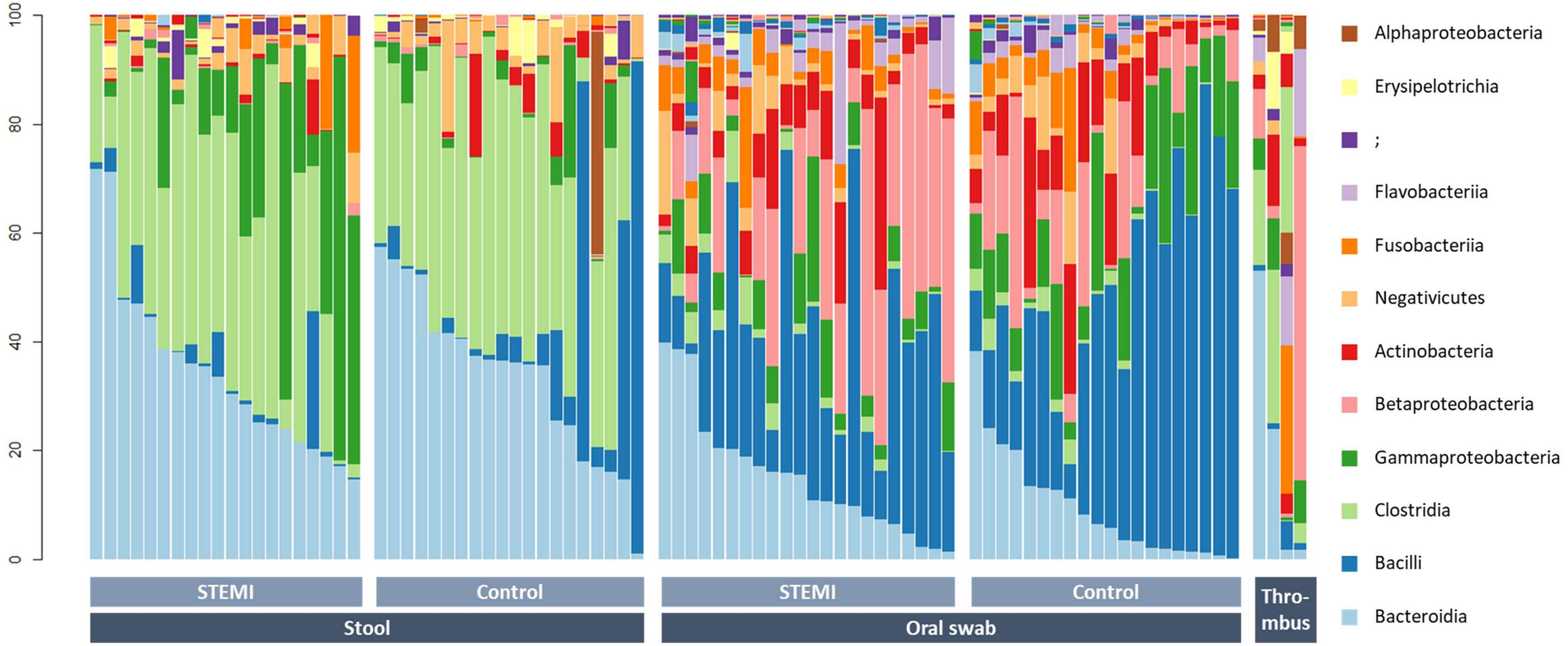

(c) Order level

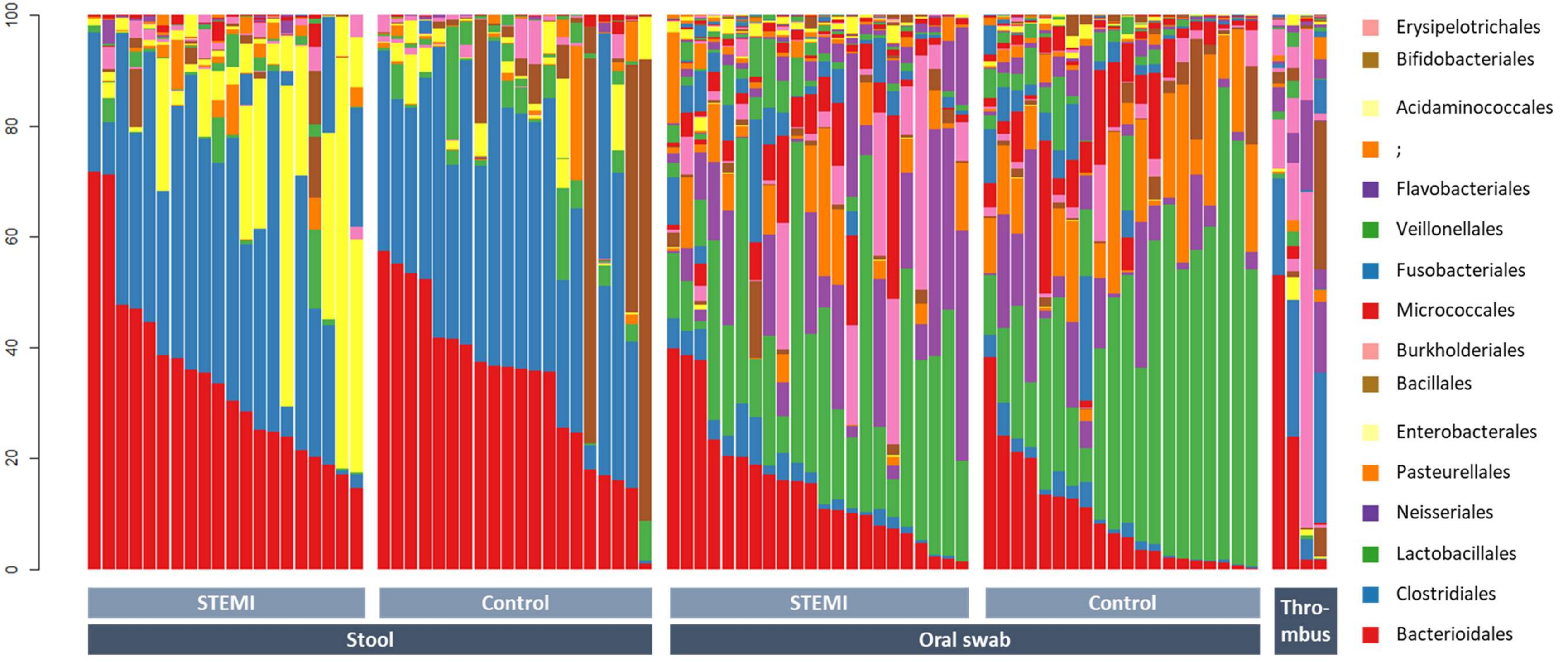

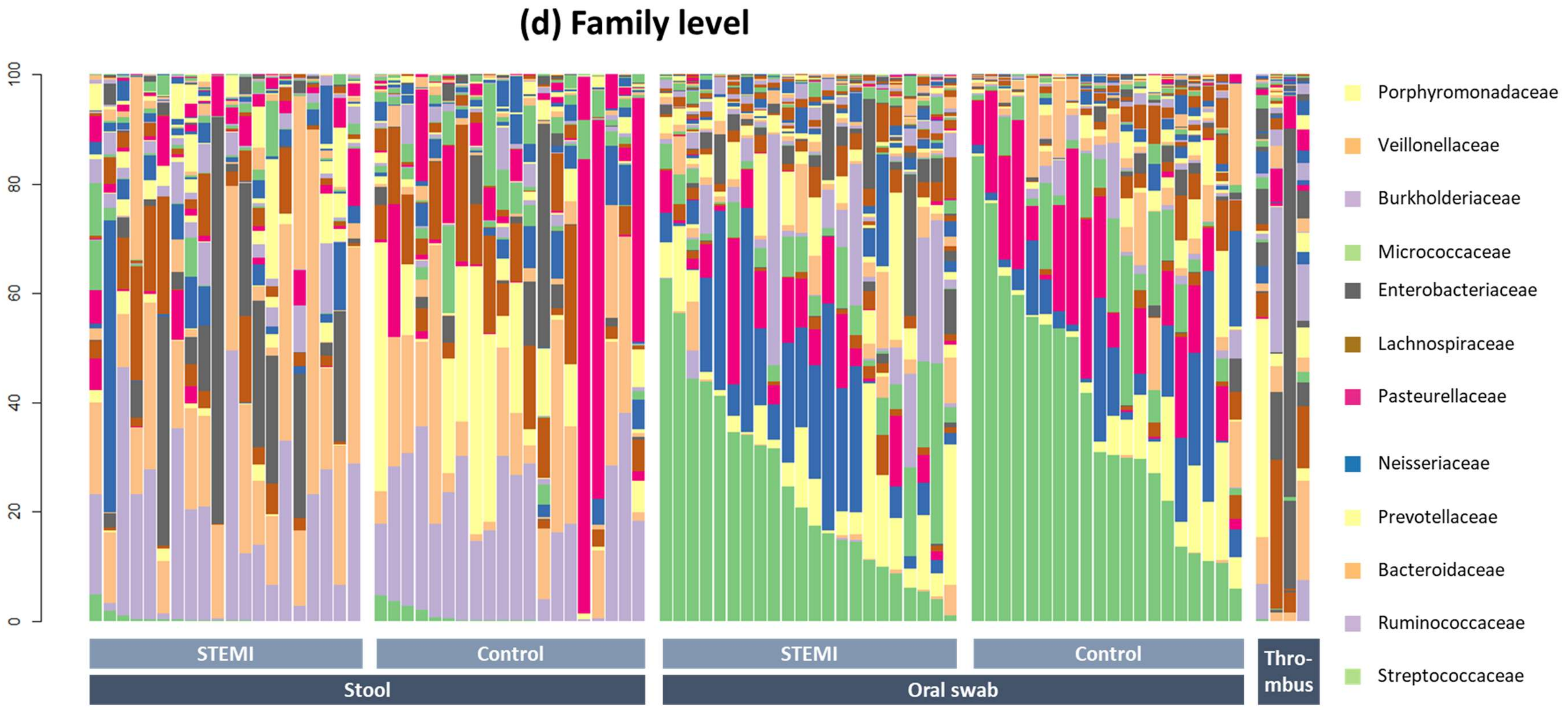

(d) Family level
0
20
40
60
80
100
STEMI
Control
STEMI
Control
Stool
Oral swab
Thro-
mbus
Porphyromonadaceae
Veillonellaceae
Burkholderiaceae
Micrococcaceae
Enterobacteriaceae
Lachnospiraceae
Pasteurellaceae
Neisseriaceae
Prevotellaceae
Bacteroidaceae
Ruminococcaceae
Streptococcaceae

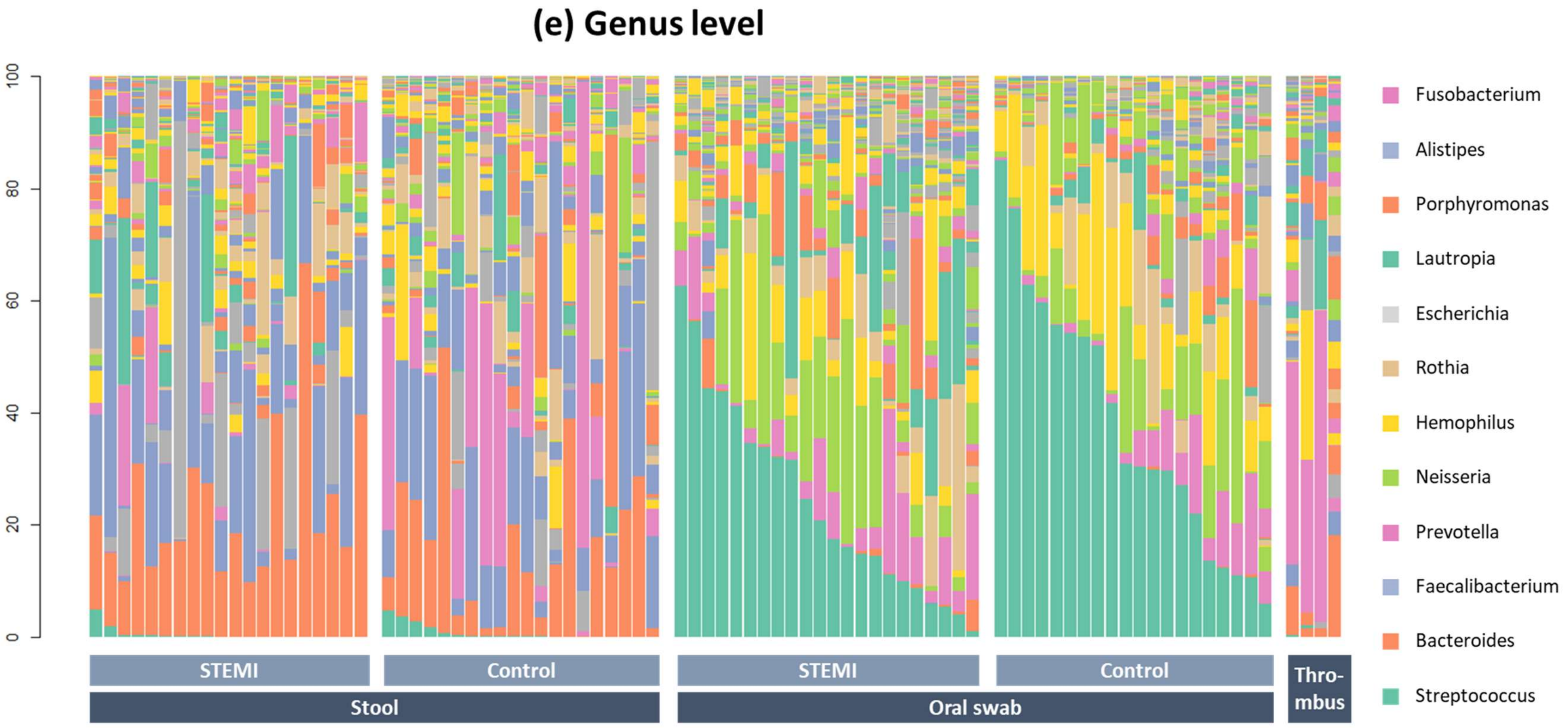
(e) Genus level
100
80
60
40
20
0
STEMI
Control
STEMI
Control
Thro-
mbus
Stool
Oral swab
Fusobacterium
Alistipes
Porphyromonas
Lautropia
Escherichia
Rothia
Hemophilus
Neisseria
Prevotella
Faecalibacterium
Bacteroides
Streptococcus

Supplementary Figure 5. Bar plot showing the side-by-side comparison of the thrombi, oral, and gut microbiota of individual patients at each taxonomic level ((a) Phylum, (b) Class, (c) Order, (d) Family, (e) Species.

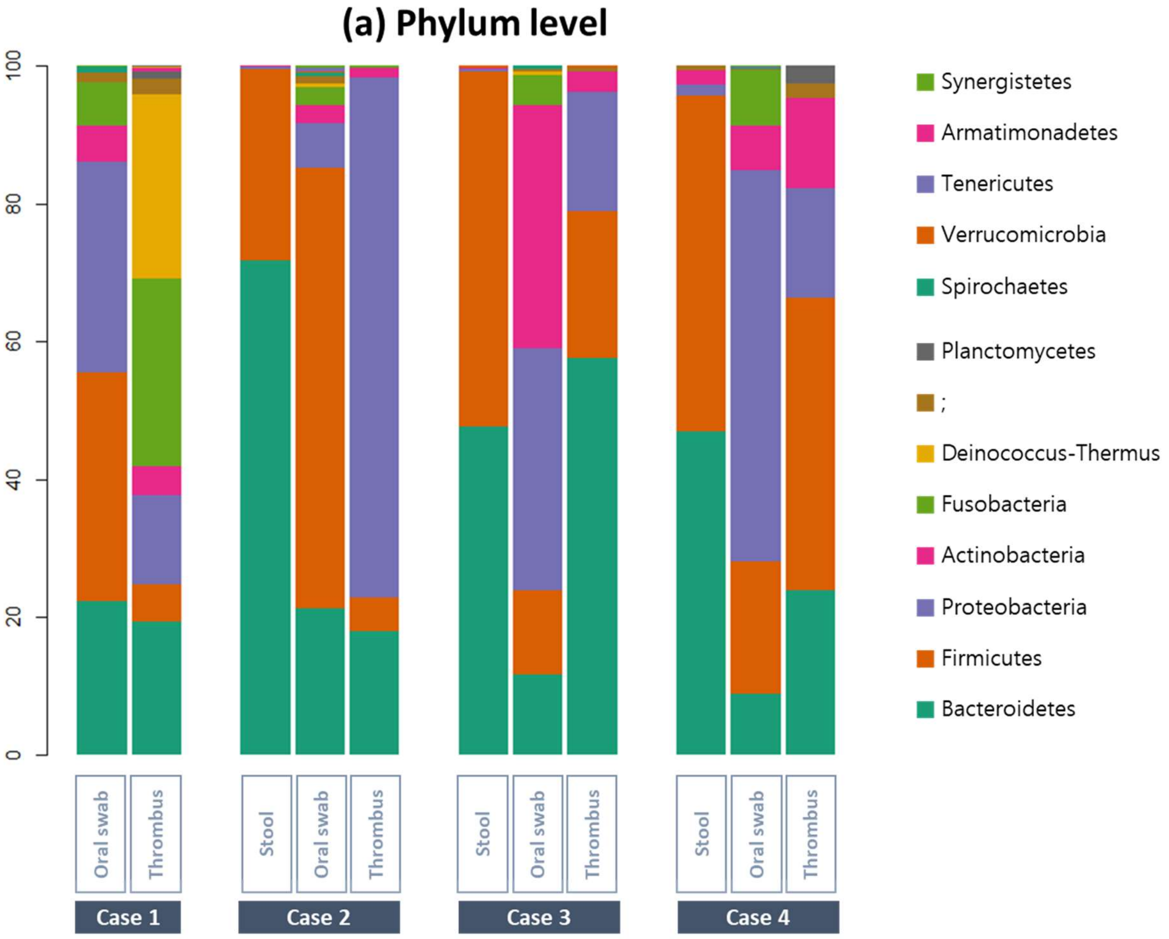

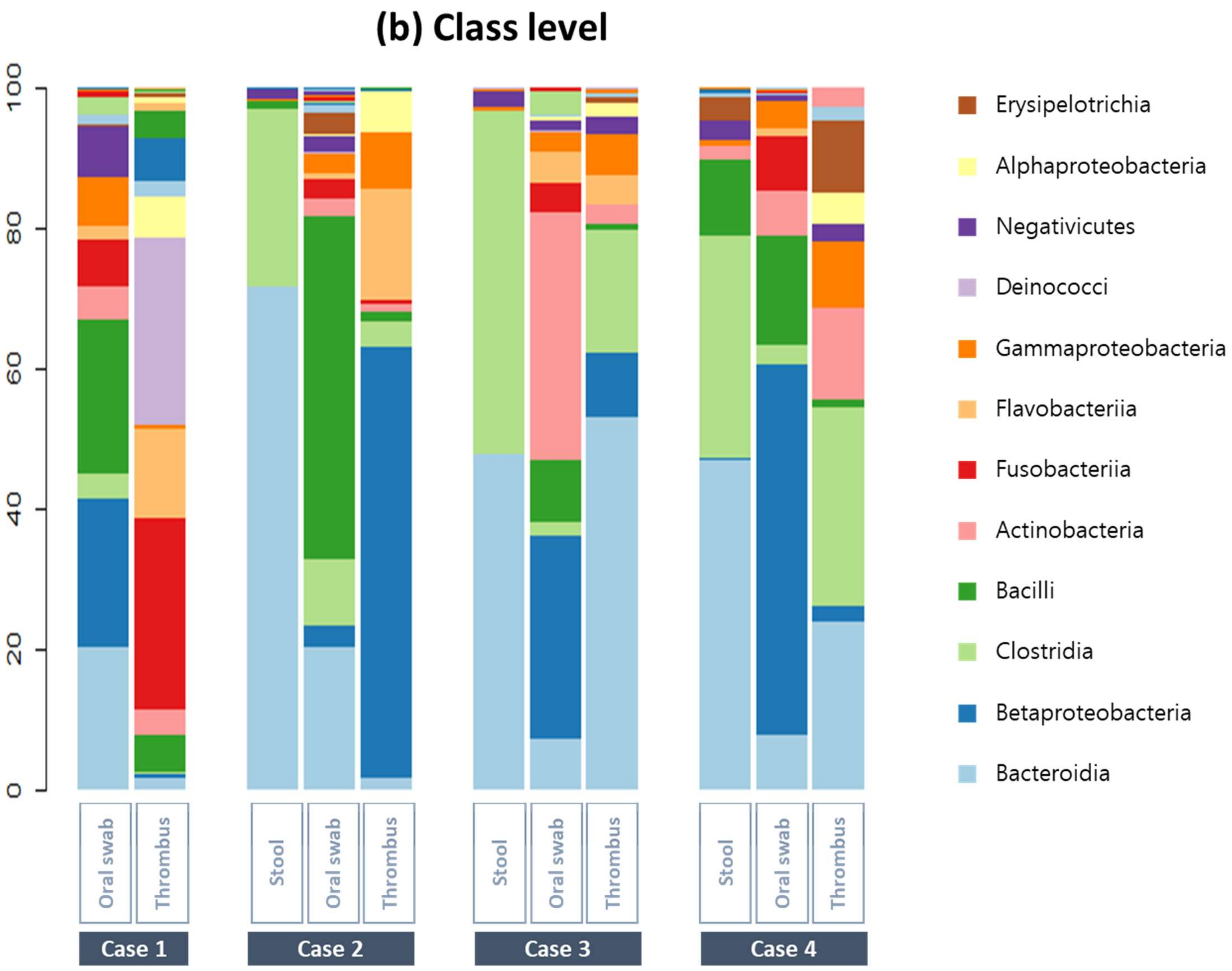

(b) Class level
0
20
40
60
80
100
Oral swab
Thrombus
Stool
Oral swab
Thrombus
Stool
Oral swab
Thrombus
Stool
Oral swab
Thrombus
Case 1
Case 2
Case 3
Case 4
Erysipelotrichia
Alphaproteobacteria
Negativicutes
Deinococci
Gammaproteobacteria
Flavobacteriia
Fusobacteriia
Actinobacteria
Bacilli
Clostridia
Betaproteobacteria
Bacteroidia

(c) Order level

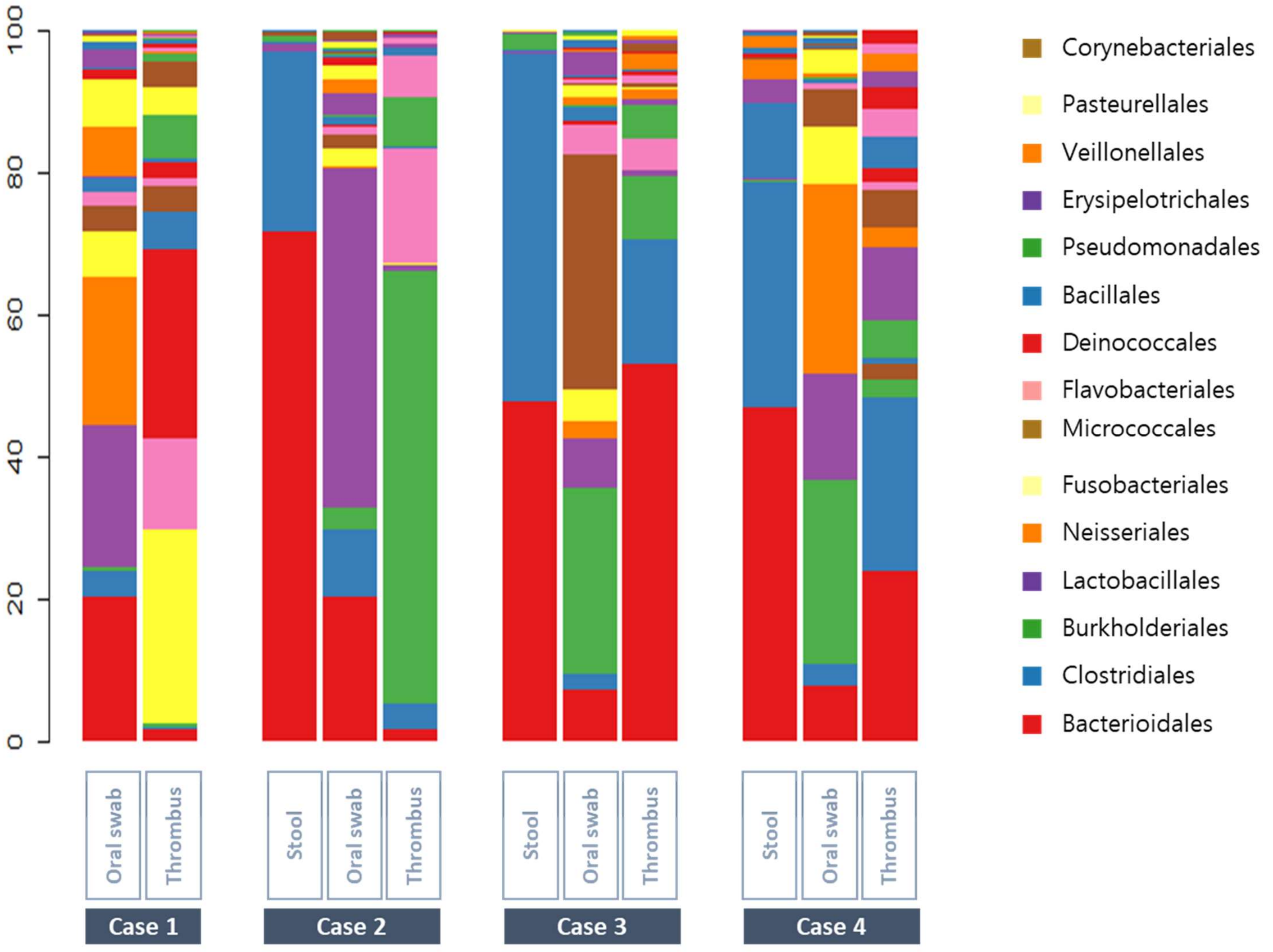

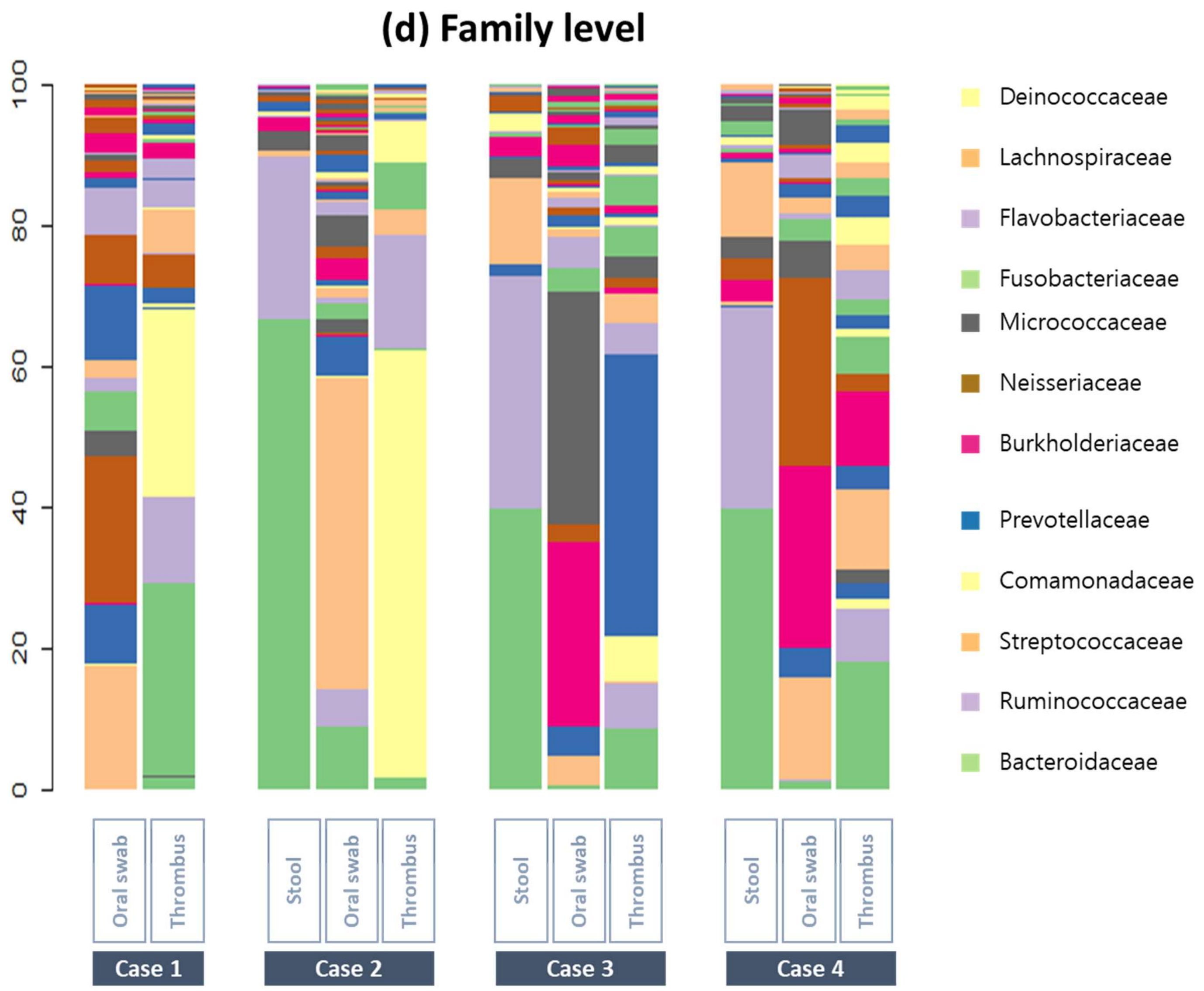

(d) Family level
100
80
60
40
20
0
Oral swab
Thrombus
Stool
Oral swab
Thrombus
Stool
Oral swab
Thrombus
Stool
Oral swab
Thrombus
Case 1
Case 2
Case 3
Case 4
Deinococcaceae
Lachnospiraceae
Flavobacteriaceae
Fusobacteriaceae
Micrococcaceae
Neisseriaceae
Burkholderiaceae
Prevotellaceae
Comamonadaceae
Streptococcaceae
Ruminococcaceae
Bacteroidaceae

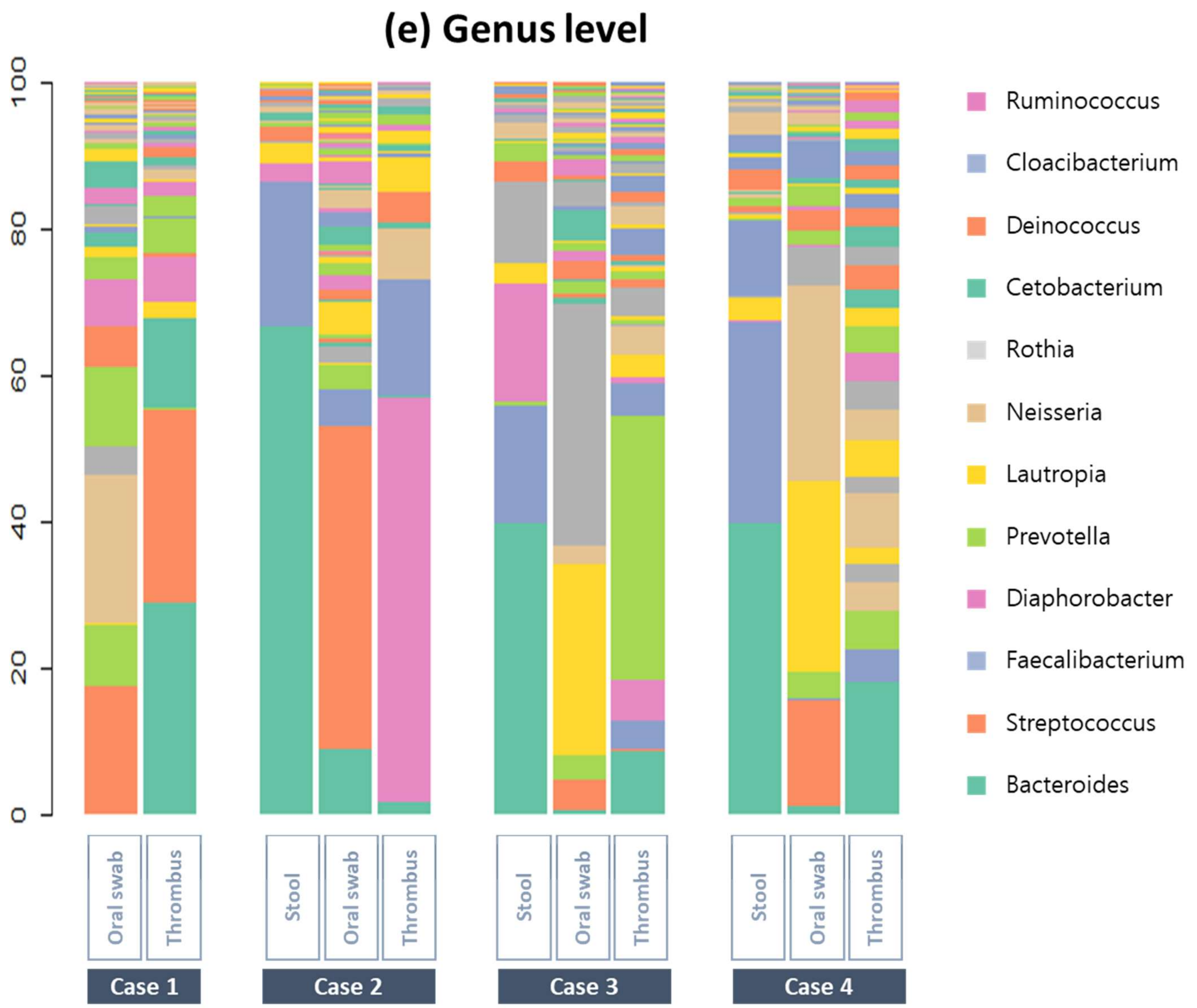
(e) Genus level
0
20
40
60
80
100
Oral swab
Thrombus
Stool
Oral swab
Thrombus
Stool
Oral swab
Thrombus
Stool
Oral swab
Thrombus
Case 1
Case 2
Case 3
Case 4
Ruminococcus
Cloacibacterium
Deinococcus
Cetobacterium
Rothia
Neisseria
Lautropia
Prevotella
Diaphorobacter
Faecalibacterium
Streptococcus
Bacteroides

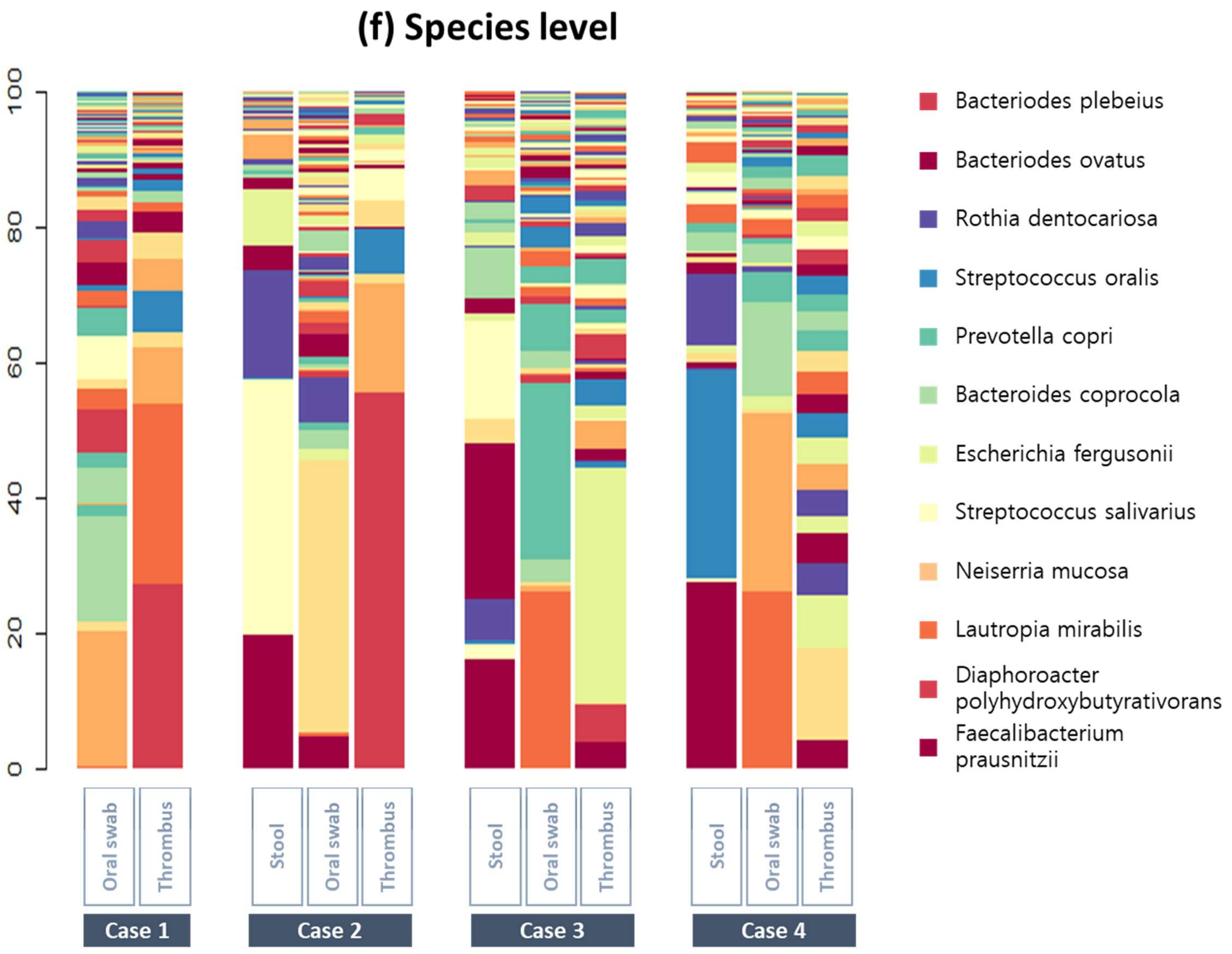

(f) Species level
0
20
40
60
80
100
Bacteriodes plebeius
Bacteriodes ovatus
Rothia dentocariosa
Streptococcus oralis
Prevotella copri
Bacteroides coprocola
Escherichia fergusonii
Streptococcus salivarius
Neiserria mucosa
Lautropia mirabilis
Diaphoroacter polyhydroxybutyrativorans
Faecalibacterium prausnitzii
Oral swab
Thrombus
Stool
Oral swab
Thrombus
Stool
Oral swab
Thrombus
Stool
Oral swab
Thrombus
Case 1
Case 2
Case 3
Case 4

Supplementary Figure 6. Beta-diversity of (a) the gut microbiome and (b) the oral microbiome using principal coordinate analysis (PCoA). OTUs of both stool and oral microbiomes showed different clustering between groups suggesting phylogenetic closeness of the microbial communities within each group.

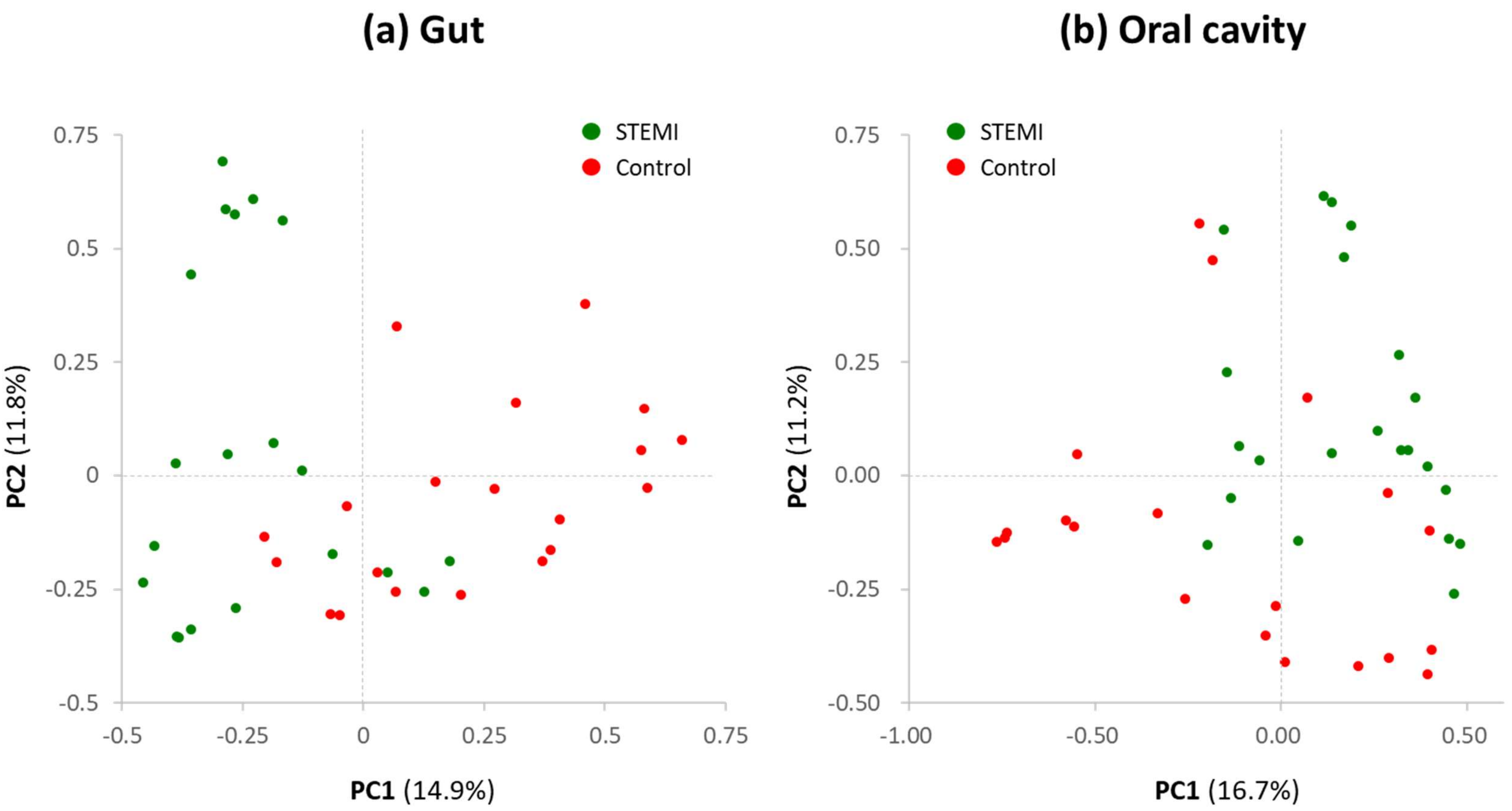

Supplementary Figure 7. Beta-diversity of (a) the gut microbiome and (b) the oral microbiome matching with individual patients with thrombi sample, using principal coordinate analysis (PCoA).

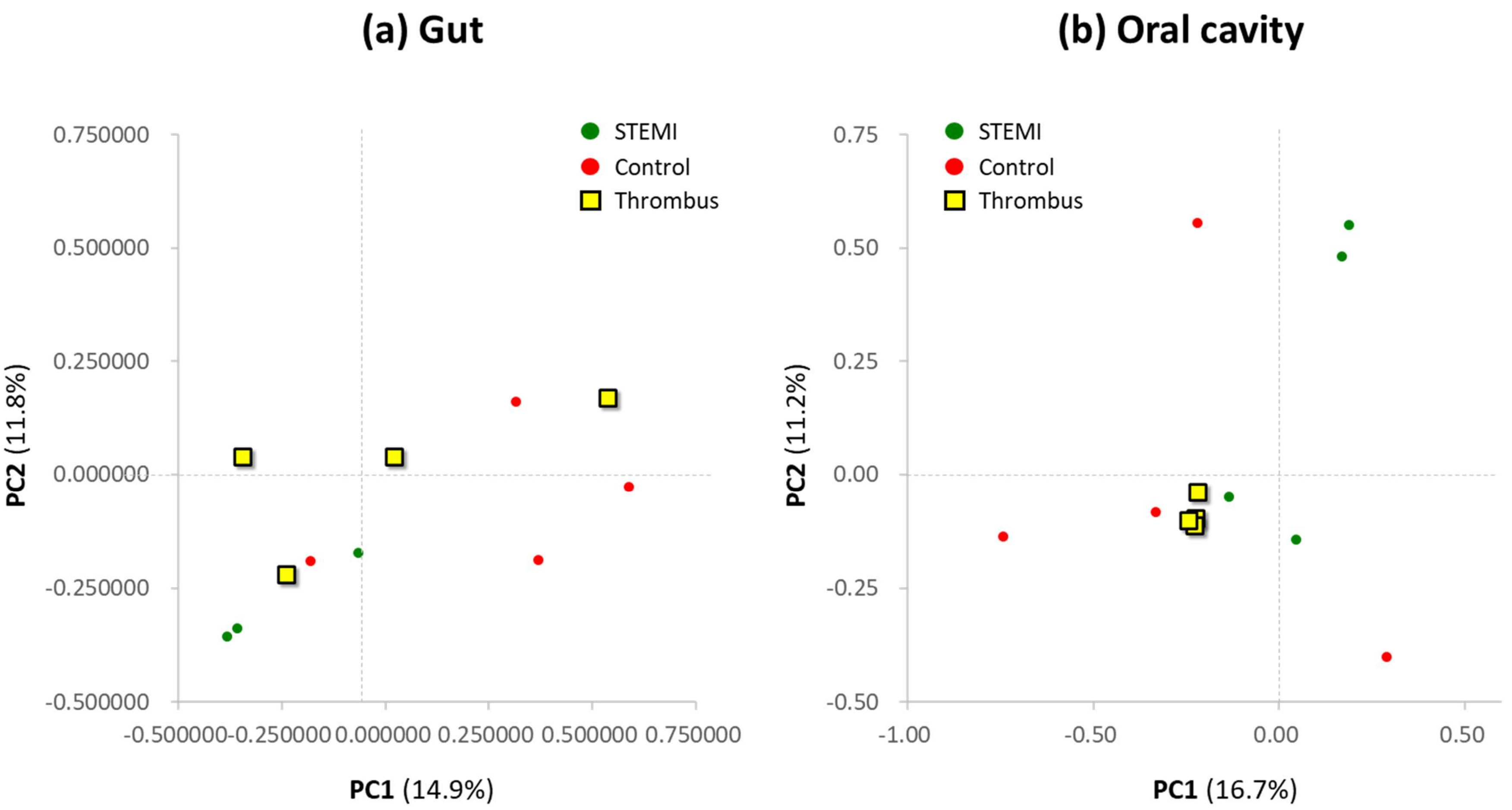

Supplementary Figure 8. Heatmap of the thrombi, oral, and gut microbiota of individual patients at the species level. Relative abundance ratios of selected biomarkers are depicted for (a) stool and thrombus, and (b) oral swab and thrombus. The blue color represents lower abundance, white color represents intermediate abundance and red represents the highest abundance.

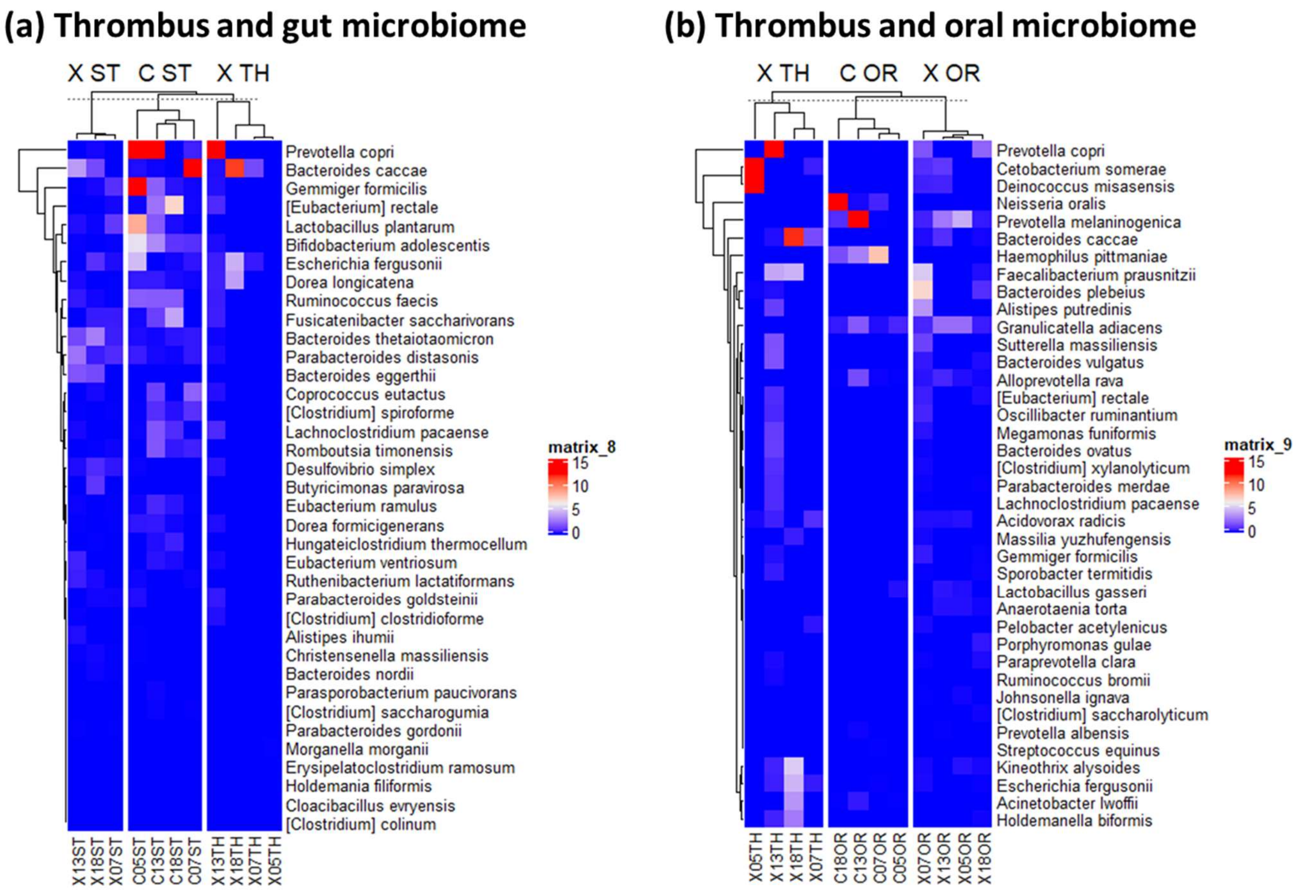

Supplementary Figure 9. Analysis of similarities (ANOSIM) was performed to compare the matrix of rank dissimilarity. The dissimilarity between the stool and thrombus samples was larger than that between the oral and thrombus samples (unweighted R=0.444 and 0.2916, respectively)

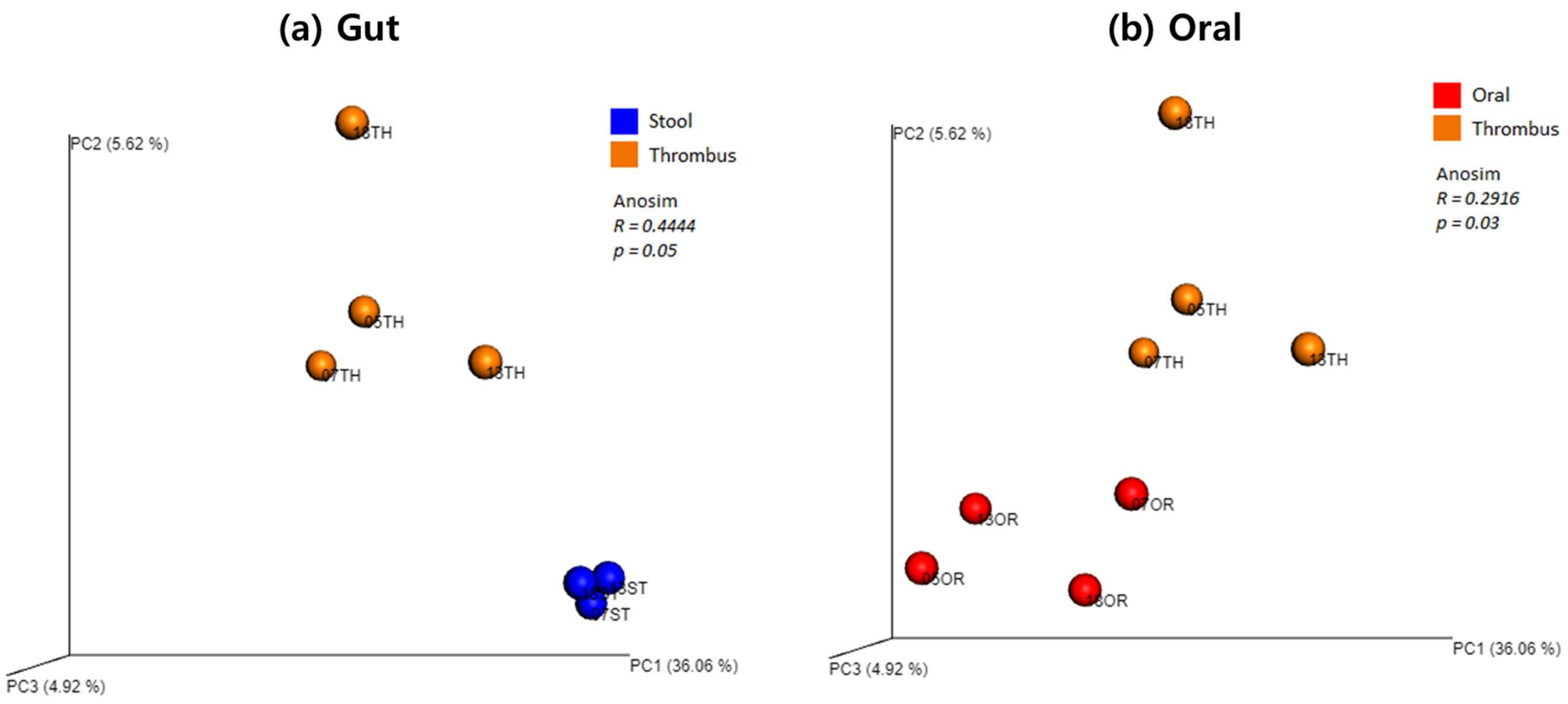

Supplementary Table 1. Full taxonomic comparative analysis of gut microbiome

Kingdom	Phylum	Class	Order	Family	Genus	STEMI	Control	Unadjusted P-value	FDR-adjusted P-value
Bacteria	Proteobacteria	Gammaproteobacteria	Enterobacterales	Enterobacteriaceae	Escherichia	10.2651%	1.4595%	0.0031	0.0292
Bacteria	Firmicutes	Clostridia	Clostridiales	Lachnospiraceae		0.8425%	4.6645%	0.0035	0.0292
Bacteria	Bacteroidetes	Bacteroidia	Bacteroidales	Tannerellaceae	Parabacteroides	3.2300%	0.6229%	0.0035	0.0292
Bacteria	Firmicutes	Clostridia	Clostridiales	Christensenellaceae	Christensenella	0.6708%	0.1739%	0.0047	0.0292
Bacteria	Firmicutes	Bacilli	Lactobacillales	Lactobacillaceae	Lactobacillus	1.1234%	2.5415%	0.0067	0.0336
Bacteria	Actinobacteria	Actinobacteria	Bifidobacteriales	Bifidobacteriaceae	Bifidobacterium	0.7869%	2.3786%	0.0239	0.0995
Bacteria	Proteobacteria	Gammaproteobacteria	Enterobacterales	Morganellaceae	Morganella	2.2715%	0.0000%	0.0402	0.1434
Bacteria	Proteobacteria	Gammaproteobacteria	Enterobacterales	Enterobacteriaceae	Citrobacter	3.7437%	0.5263%	0.0564	0.1764
Bacteria	Bacteroidetes	Bacteroidia	Bacteroidales	Bacteroidaceae	Bacteroides	22.2797%	15.0051%	0.0675	0.1875
Bacteria	Fusobacteria	Fusobacteriia	Fusobacteriales	Fusobacteriaceae	Fusobacterium	2.5214%	0.0139%	0.0922	0.2304
Bacteria	Firmicutes	Clostridia	Clostridiales	Ruminococcaceae	Ruminococcus	1.5151%	1.7450%	0.1167	0.2454
Bacteria	Proteobacteria	Gammaproteobacteria	Pasteurellales	Pasteurellaceae	Haemophilus	0.9546%	1.5414%	0.1178	0.2454
Bacteria	Bacteroidetes	Bacteroidia	Bacteroidales	Odoribacteraceae	Butyricimonas	0.8048%	0.3148%	0.1539	0.2960
Bacteria	Firmicutes	Clostridia	Clostridiales	Lachnospiraceae	Blautia	0.9981%	0.8715%	0.2110	0.3620
Bacteria	Firmicutes	Negativicutes	Acidaminococcales	Acidaminococcaceae	Phascolarctobacterium	2.0049%	2.1998%	0.2172	0.3620
Unassigned	Unassigned	Unassigned	Unassigned	Unassigned	Unassigned	1.2989%	0.6593%	0.3301	0.5012
Bacteria	Firmicutes	Clostridia	Clostridiales	Eubacteriaceae	Eubacterium	0.9254%	1.4442%	0.3408	0.5012
Bacteria	Firmicutes	Clostridia	Clostridiales	Defluviitaleaceae	Vallitalea	1.7058%	0.8457%	0.3752	0.5212
Bacteria	Firmicutes	Clostridia	Clostridiales	Oscillospiraceae	Oscillibacter	1.9302%	2.0299%	0.4093	0.5329
Bacteria	Firmicutes	Clostridia	Clostridiales	Gracilibacteraceae	Gracilibacter	0.8388%	0.3469%	0.4383	0.5329
Bacteria	Firmicutes	Clostridia	Clostridiales	Hungateiclostridiaceae	Anaerobacterium	0.8107%	0.0715%	0.4555	0.5329
Bacteria	Firmicutes	Erysipelotrichia	Erysipelotrichales	Erysipelotrichaceae	Holdemanella	0.7048%	0.7657%	0.4698	0.5329
Bacteria	Bacteroidetes	Bacteroidia	Bacteroidales	Rikenellaceae	Alistipes	5.8356%	3.1278%	0.4903	0.5329
Bacteria	Firmicutes	Clostridia	Clostridiales	Ruminococcaceae	Faecalibacterium	16.1796%	14.2183%	0.3132	0.6364
Bacteria	Bacteroidetes	Bacteroidia	Bacteroidales	Porphyromonadaceae	Porphyromonas	0.6308%	0.6787%	0.8887	0.8887
Bacteria	Proteobacteria	Deltaproteobacteria	Desulfovibrionales	Desulfovibrionaceae	Desulfovibrio	0.2923%	0.0838%	0.0012	NA
Bacteria	Bacteroidetes	Bacteroidia	Bacteroidales	Prevotellaceae	Prevotella	0.3867%	11.3933%	0.0047	NA
Bacteria	Firmicutes	Clostridia	Clostridiales	Lachnospiraceae	Dorea	0.1000%	0.2742%	0.0051	NA
Bacteria	Firmicutes	Clostridia	Clostridiales	Ruminococcaceae	Ruthenibacterium	0.1952%	0.0277%	0.0065	NA
Bacteria	Firmicutes	Clostridia	Clostridiales	Lachnospiraceae	Coprococcus	0.6192%	1.1393%	0.0178	NA
Bacteria	Firmicutes	Erysipelotrichia	Erysipelotrichales	Erysipelotrichaceae	Holdemania	0.0000%	0.0040%	0.0198	NA
Bacteria	Firmicutes	Clostridia	Clostridiales	Lachnospiraceae	Fusicatenibacter	0.2271%	0.5500%	0.0231	NA
Bacteria	Firmicutes	Clostridia	Clostridiales	Ruminococcaceae	Gemmiger	0.2292%	1.6986%	0.0326	NA
Bacteria	Firmicutes	Clostridia	Clostridiales	Peptostreptococcaceae	Romboutsia	0.1579%	0.6385%	0.0356	NA
Bacteria	Firmicutes	Clostridia	Clostridiales	Hungateiclostridiaceae	Hungateiclostridium	0.0677%	0.1065%	0.0386	NA
Bacteria	Firmicutes	Clostridia	Clostridiales	Lachnospiraceae	Parasporobacterium	0.0000%	0.0118%	0.0402	NA
Bacteria	Firmicutes	Clostridia	Clostridiales	Lachnospiraceae	Tyzzerella	0.0000%	0.0035%	0.0402	NA
Bacteria	Synergistetes	Synergistia	Synergistales	Synergistaceae	Cloacibacillus	0.0416%	0.0026%	0.0521	NA
Bacteria	Firmicutes	Clostridia	Clostridiales	Ruminococcaceae	Agathobaculum	0.0175%	0.1270%	0.0622	NA
Bacteria	Proteobacteria	Gammaproteobacteria	Enterobacterales	Morganellaceae	Providencia	0.1732%	0.0000%	0.0806	NA
Bacteria	Actinobacteria	Actinobacteria	Corynebacteriales	Corynebacteriaceae	Corynebacterium	0.0190%	0.0000%	0.0806	NA
Bacteria	Proteobacteria	Gammaproteobacteria	Enterobacterales	Enterobacteriaceae	Raoultella	0.0116%	0.0000%	0.0806	NA
Bacteria	Proteobacteria	Gammaproteobacteria	Enterobacterales	Enterobacteriaceae	Klebsiella	0.0029%	0.0000%	0.0806	NA

Bacteria	Proteobacteria	Gammaproteobacteria	Enterobacterales	Enterobacteriaceae	Enterobacter	0.0027%	0.0000%	0.0806	NA
Bacteria	Firmicutes	Tissierellia	Tissierellales	Peptoniphilaceae	Peptoniphilus	0.0015%	0.0000%	0.0806	NA
Bacteria	Firmicutes	Bacilli	Lactobacillales	Lactobacillaceae	Pediococcus	0.0000%	0.0043%	0.0806	NA
Bacteria	Firmicutes	Clostridia	Clostridiales	Ruminococcaceae	Subdoligranulum	0.0000%	0.0024%	0.0806	NA
Bacteria	Firmicutes	Clostridia	Clostridiales	Ruminococcaceae	Harryflintia	0.0074%	0.0007%	0.0816	NA
Bacteria	Bacteroidetes	Bacteroidia	Bacteroidales	Prevotellaceae	Paraprevotella	0.3008%	0.2271%	0.0824	NA
Bacteria	Firmicutes	Clostridia	Clostridiales		Natranaerovirga	0.0063%	0.3883%	0.0895	NA
Bacteria	Actinobacteria	Coriobacteriia	Eggerthellales	Eggerthellaceae	Slackia	0.0021%	0.0346%	0.0895	NA
Bacteria	Firmicutes	Clostridia	Clostridiales	Ruminococcaceae	Acetivibrio	0.0045%	0.0855%	0.0986	NA
Bacteria	Proteobacteria	Betaproteobacteria	Burkholderiales	Burkholderiaceae	Lautropia	0.0030%	0.0011%	0.1072	NA
Bacteria	Bacteroidetes	Bacteroidia	Bacteroidales	Barnesiellaceae	Barnesiella	0.2736%	1.4190%	0.1090	NA
Bacteria	Firmicutes	Clostridia	Clostridiales	Lachnospiraceae	Lachnospira	0.1088%	0.1004%	0.1121	NA
Bacteria	Firmicutes	Clostridia	Clostridiales	Lachnospiraceae	Roseburia	0.2200%	0.2552%	0.1181	NA
Bacteria	Firmicutes	Clostridia	Clostridiales	Clostridiaceae	Butyricicoccus	0.0198%	0.0200%	0.1262	NA
Bacteria	Firmicutes	Clostridia	Clostridiales	Lachnospiraceae	Pseudobutyrivibrio	0.0090%	0.0357%	0.1290	NA
Bacteria	Firmicutes	Clostridia	Clostridiales	Clostridiaceae	Caloramator	0.1799%	0.0703%	0.1532	NA
Bacteria	Proteobacteria	Gammaproteobacteria	Enterobacterales	Morganellaceae	Proteus	0.5010%	0.0000%	0.1626	NA
Bacteria	Firmicutes	Clostridia	Clostridiales	Lachnospiraceae	Abyssivirga	0.1619%	0.0000%	0.1626	NA
Bacteria	Firmicutes	Tissierellia	Tissierellales	Tissierellaceae		0.1566%	0.0000%	0.1626	NA
Bacteria	Bacteroidetes	Bacteroidia	Bacteroidales	Prevotellaceae	Marseilla	0.0110%	0.0000%	0.1626	NA
Bacteria	Tenericutes	Mollicutes	Acholeplasmatales	Acholeplasmataceae	Acholeplasma	0.0037%	0.0000%	0.1626	NA
Bacteria	Proteobacteria	Gammaproteobacteria	Enterobacterales	Enterobacteriaceae	Shigella	0.0024%	0.0000%	0.1626	NA
Bacteria	Firmicutes	Clostridia	Clostridiales	Peptostreptococcaceae	Peptostreptococcus	0.0007%	0.0000%	0.1626	NA
Bacteria	Firmicutes	Tissierellia	Tissierellales	Peptoniphilaceae	Parvimonas	0.0007%	0.0000%	0.1626	NA
Bacteria	Bacteroidetes	Flavobacteriia	Flavobacteriales	Flavobacteriaceae	Cloacibacterium	0.0007%	0.0000%	0.1626	NA
Bacteria	Proteobacteria	Gammaproteobacteria	Cardiobacteriales	Cardiobacteriaceae	Cardiobacterium	0.0006%	0.0000%	0.1626	NA
Bacteria	Actinobacteria	Coriobacteriia	Coriobacteriales	Atopobiaceae	Olsenella	0.0000%	0.0021%	0.1626	NA
Bacteria	Firmicutes	Clostridia	Clostridiales	Peptostreptococcaceae	Clostridioides	0.0000%	0.0045%	0.1626	NA
Bacteria	Firmicutes	Erysipelotrichia	Erysipelotrichales	Erysipelotrichaceae	Faecalibaculum	0.0000%	0.0006%	0.1626	NA
Bacteria	Proteobacteria	Alphaproteobacteria	Rhodospirillales	Rhodospirillaceae	Fodinicurvata	0.0000%	0.0055%	0.1626	NA
Bacteria	Proteobacteria	Alphaproteobacteria	Rhodospirillales	Rhodospirillaceae	Novispirillum	0.0000%	2.1505%	0.1626	NA
Bacteria	Firmicutes	Clostridia	Clostridiales	Lachnospiraceae	Lachnoclostridium	0.2231%	0.5101%	0.1635	NA
Bacteria	Firmicutes	Clostridia	Clostridiales	Lachnospiraceae	Murimonas	0.0056%	0.0207%	0.1663	NA
Bacteria	Firmicutes	Clostridia	Clostridiales	Lachnospiraceae	Kineothrix	0.1270%	0.2300%	0.1717	NA
Bacteria	Firmicutes	Erysipelotrichia	Erysipelotrichales	Erysipelotrichaceae	Catenibacterium	0.3080%	0.2131%	0.1760	NA
Bacteria	Firmicutes	Clostridia	Clostridiales	Ruminococcaceae	Anaeromassilibacillus	0.0267%	0.0374%	0.1932	NA
Bacteria	Proteobacteria	Epsilonproteobacteria	Campylobacterales	Campylobacteraceae	Campylobacter	0.0008%	0.0016%	0.1955	NA
Bacteria	Firmicutes	Clostridia	Clostridiales	Clostridiales_Family_XIII._Incertae_Sedis	Emergencia	0.0018%	0.0071%	0.2129	NA
Bacteria	Firmicutes	Clostridia	Clostridiales		Intestinimonas	0.4951%	0.0279%	0.2276	NA
Bacteria	Bacteroidetes	Bacteroidia	Bacteroidales	Odoribacteraceae	Odoribacter	0.3983%	0.2328%	0.2321	NA
Bacteria	Firmicutes	Clostridia	Clostridiales	Lachnospiraceae	Lachnobacterium	0.0034%	0.0107%	0.2439	NA
Bacteria	Firmicutes	Negativicutes	Veillonellales	Veillonellaceae	Dialister	0.6245%	1.7390%	0.2506	NA
Bacteria	Actinobacteria	Coriobacteriia	Eggerthellales	Eggerthellaceae	Eggerthella	0.0906%	0.0374%	0.2565	NA
Bacteria	Firmicutes	Clostridia	Clostridiales	Lachnospiraceae	Faecalicatena	0.0094%	0.0017%	0.2592	NA
Bacteria	Firmicutes	Bacilli	Lactobacillales	Streptococcaceae	Streptococcus	0.4133%	0.7426%	0.2597	NA
Bacteria	Firmicutes	Bacilli	Lactobacillales	Leuconostocaceae	Leuconostoc	0.0044%	0.0039%	0.2614	NA
Bacteria	Firmicutes	Negativicutes	Veillonellales	Veillonellaceae	Veillonella	0.2519%	0.3478%	0.2637	NA
Bacteria	Proteobacteria	Gammaproteobacteria	Pseudomonadales	Pseudomonadaceae	Pseudomonas	0.3030%	0.0018%	0.2753	NA

Bacteria	Firmicutes	Clostridia	Clostridiales	Lachnospiraceae	Anaerotaenia	0.0369%	0.0332%	0.2782	NA
Bacteria	Firmicutes	Clostridia	Clostridiales	Ruminococcaceae	Acetanaerobacterium	0.2209%	0.3325%	0.3095	NA
Bacteria	Firmicutes	Clostridia	Clostridiales	Lachnospiraceae	Anaerotignum	0.0048%	0.0241%	0.3170	NA
Bacteria	Firmicutes	Clostridia	Clostridiales	Eubacteriaceae	Rhabdanaerobium	0.0064%	0.0022%	0.3236	NA
Bacteria	Firmicutes	Clostridia	Clostridiales	Ruminococcaceae	Papillibacter	0.0263%	0.0019%	0.3273	NA
Bacteria	Proteobacteria	Betaproteobacteria	Burkholderiales	Sutterellaceae	Duodenibacillus	0.0020%	0.0350%	0.3273	NA
Bacteria	Firmicutes	Erysipelotrichia	Erysipelotrichales	Erysipelotrichaceae	Erysipelatoclostridium	0.1159%	0.4159%	0.3350	NA
Bacteria	Firmicutes	Bacilli	Bacillales	Planococcaceae	Sporosarcina	0.1462%	0.0000%	0.3421	NA
Bacteria	Firmicutes	Bacilli	Bacillales	Planococcaceae	Solibacillus	0.0912%	0.0000%	0.3421	NA
Bacteria	Firmicutes	Bacilli	Bacillales	Bacillaceae	Lysinibacillus	0.0860%	0.0000%	0.3421	NA
Bacteria	Firmicutes	Clostridia	Clostridiales	Clostridiaceae	Alkaliphilus	0.0546%	0.0000%	0.3421	NA
Bacteria	Actinobacteria	Actinobacteria	Streptomycetales	Streptomycetaceae	Streptomyces	0.0230%	0.0000%	0.3421	NA
Bacteria	Bacteroidetes	Sphingobacteriia	Sphingobacteriales	Sphingobacteriaceae	Solitalea	0.0116%	0.0000%	0.3421	NA
Archaea	Euryarchaeota	Thermoplasmata	Methanomassiliicoccales	Methanomassiliicoccaceae	Methanomassiliicoccus	0.0090%	0.0000%	0.3421	NA
Bacteria	Bacteroidetes	Bacteroidia	Bacteroidales	Muribaculaceae	Muribaculum	0.0045%	0.0000%	0.3421	NA
Bacteria	Firmicutes	Clostridia	Thermoanaerobacterales	Thermoanaerobacteraceae	Tepidanaerobacter	0.0042%	0.0000%	0.3421	NA
Bacteria	Fusobacteria	Fusobacteriia	Fusobacteriales	Fusobacteriaceae	Cetobacterium	0.0034%	0.0000%	0.3421	NA
Bacteria	Bacteroidetes	Bacteroidia	Bacteroidales	Paludibacteraceae	Paludibacter	0.0031%	0.0000%	0.3421	NA
Bacteria	Firmicutes	Erysipelotrichia	Erysipelotrichales	Erysipelotrichaceae	Breznakia	0.0029%	0.0000%	0.3421	NA
Bacteria	Bacteroidetes	Flavobacteriia	Flavobacteriales	Flavobacteriaceae	Flavobacterium	0.0027%	0.0000%	0.3421	NA
Bacteria	Deinococcus.Thermus	Deinococci	Deinococcales	Deinococcaceae	Deinococcus	0.0025%	0.0000%	0.3421	NA
Bacteria	Firmicutes	Clostridia	Clostridiales	Lachnospiraceae	Cellulosilyticum	0.0024%	0.0000%	0.3421	NA
Bacteria	Proteobacteria	Gammaproteobacteria	Enterobacterales	Erwiniaceae	Pantoea	0.0023%	0.0000%	0.3421	NA
Bacteria	Firmicutes	Tissierellia			Sedimentibacter	0.0020%	0.0000%	0.3421	NA
Bacteria	Firmicutes	Clostridia	Clostridiales	Peptococcaceae	Peptococcus	0.0018%	0.0000%	0.3421	NA
Bacteria	Proteobacteria	Deltaproteobacteria	Desulfovibrionales	Desulfovibrionaceae	Mailhella	0.0012%	0.0000%	0.3421	NA
Bacteria	Firmicutes	Bacilli	Bacillales	Thermoactinomycetaceae	Marinithermofilum	0.0012%	0.0000%	0.3421	NA
Bacteria	Proteobacteria	Gammaproteobacteria	Enterobacterales	Enterobacteriaceae	Kluyvera	0.0012%	0.0000%	0.3421	NA
Bacteria	Firmicutes	Clostridia	Clostridiales	Clostridiaceae	Brassicibacter	0.0011%	0.0000%	0.3421	NA
Bacteria	Firmicutes	Clostridia	Clostridiales	Peptococcaceae	Desulfohalotomaculum	0.0010%	0.0000%	0.3421	NA
Bacteria	Proteobacteria	Gammaproteobacteria	Oceanospirillales	Endozoicomonadaceae	Endozoicomonas	0.0010%	0.0000%	0.3421	NA
Bacteria	Proteobacteria	Deltaproteobacteria	Myxococcales	Polyangiaceae	Chondromyces	0.0009%	0.0000%	0.3421	NA
Bacteria	Firmicutes	Clostridia	Clostridiales	Hungateiclostridiaceae	Mageeibacillus	0.0009%	0.0000%	0.3421	NA
Bacteria	Firmicutes	Tissierellia			Dethiosulfatibacter	0.0009%	0.0000%	0.3421	NA
Bacteria	Proteobacteria	Gammaproteobacteria	Enterobacterales	Enterobacteriaceae	Salmonella	0.0008%	0.0000%	0.3421	NA
Bacteria	Actinobacteria	Actinobacteria	Kineosporiales	Kineosporiaceae	Kineococcus	0.0008%	0.0000%	0.3421	NA
Bacteria	Firmicutes	Bacilli	Lactobacillales	Aerococcaceae	Abiotrophia	0.0007%	0.0000%	0.3421	NA
Bacteria	Firmicutes	Bacilli	Bacillales		Exiguobacterium	0.0007%	0.0000%	0.3421	NA
Bacteria	Firmicutes	Bacilli	Bacillales	Staphylococcaceae	Staphylococcus	0.0006%	0.0000%	0.3421	NA
Bacteria	Bacteroidetes	Chitinophagia	Chitinophagales	Chitinophagaceae	Ferruginibacter	0.0004%	0.0000%	0.3421	NA
Bacteria	Fusobacteria	Fusobacteriia	Fusobacteriales	Leptotrichiaceae	Leptotrichia	0.0004%	0.0000%	0.3421	NA
Bacteria	Synergistetes	Synergistia	Synergistales	Synergistaceae	Fretibacterium	0.0004%	0.0000%	0.3421	NA
Bacteria	Firmicutes	Erysipelotrichia	Erysipelotrichales	Erysipelotrichaceae	Solobacterium	0.0003%	0.0000%	0.3421	NA
Bacteria	Proteobacteria	Deltaproteobacteria	Desulfuromonadales	Desulfuromonadaceae	Pelobacter	0.0003%	0.0000%	0.3421	NA
Bacteria	Actinobacteria	Actinobacteria	Corynebacteriales	Mycobacteriaceae	Mycolicibacterium	0.0002%	0.0000%	0.3421	NA
Bacteria	Bacteroidetes	Chitinophagia	Chitinophagales	Chitinophagaceae	Flavitalea	0.0002%	0.0000%	0.3421	NA
Bacteria	Proteobacteria	Alphaproteobacteria	Rhizobiales		Nordella	0.0002%	0.0000%	0.3421	NA
Bacteria	Proteobacteria	Alphaproteobacteria	Rhizobiales	Phyllobacteriaceae	Roseitalea	0.0002%	0.0000%	0.3421	NA

Bacteria	Proteobacteria	Alphaproteobacteria	Sphingomonadales	Erythrobacteraceae	Altererythrobacter	0.0002%	0.0000%	0.3421	NA
Bacteria	Firmicutes	Clostridia	Clostridiales	Eubacteriaceae	Pseudoramibacter	0.0002%	0.0000%	0.3421	NA
Bacteria	Proteobacteria	Betaproteobacteria	Burkholderiales	Comamonadaceae	Acidovorax	0.0002%	0.0000%	0.3421	NA
Bacteria	Proteobacteria	Betaproteobacteria	Burkholderiales	Comamonadaceae	Kinneretia	0.0001%	0.0000%	0.3421	NA
Bacteria	Actinobacteria	Actinobacteria	Actinomycetales	Actinomycetaceae	Actinobaculum	0.0000%	0.0002%	0.3421	NA
Bacteria	Actinobacteria	Actinobacteria	Propionibacteriales	Propionibacteriaceae	Propionibacterium	0.0000%	0.0005%	0.3421	NA
Bacteria	Bacteroidetes	Bacteroidia	Bacteroidales	Bacteroidaceae	Mediterranea	0.0000%	0.1853%	0.3421	NA
Bacteria	Bacteroidetes	Flavobacteriia	Flavobacteriales	Flavobacteriaceae	Aurantiacicella	0.0000%	0.0053%	0.3421	NA
Bacteria	Firmicutes	Bacilli	Bacillales	Bacillaceae	Cerasibacillus	0.0000%	0.0008%	0.3421	NA
Bacteria	Firmicutes	Bacilli	Bacillales	Paenibacillaceae	Paenibacillus	0.0000%	0.0038%	0.3421	NA
Bacteria	Firmicutes	Clostridia	Clostridiales			0.0000%	0.0013%	0.3421	NA
Bacteria	Firmicutes	Clostridia	Clostridiales	Clostridiaceae	Beduini	0.0000%	0.0014%	0.3421	NA
Bacteria	Firmicutes	Clostridia	Clostridiales	Clostridiaceae	Falcatimonas	0.0000%	0.0011%	0.3421	NA
Bacteria	Firmicutes	Clostridia	Clostridiales	Eubacteriaceae	Irregularibacter	0.0000%	0.0013%	0.3421	NA
Bacteria	Firmicutes	Clostridia	Clostridiales	Lachnospiraceae	Anaerobium	0.0000%	0.0010%	0.3421	NA
Bacteria	Firmicutes	Clostridia	Clostridiales	Lachnospiraceae	Anaerocolumna	0.0000%	0.0420%	0.3421	NA
Bacteria	Firmicutes	Clostridia	Clostridiales	Lachnospiraceae	Faecalimonas	0.0000%	0.0008%	0.3421	NA
Bacteria	Firmicutes	Clostridia	Clostridiales	Lachnospiraceae	Mobilitalea	0.0000%	0.0074%	0.3421	NA
Bacteria	Firmicutes	Clostridia	Clostridiales	Lachnospiraceae	Muricomes	0.0000%	0.0003%	0.3421	NA
Bacteria	Firmicutes	Clostridia	Clostridiales	Lachnospiraceae	Oribacterium	0.0000%	0.0004%	0.3421	NA
Bacteria	Firmicutes	Clostridia	Clostridiales	Lachnospiraceae	Sellimonas	0.0000%	0.0013%	0.3421	NA
Bacteria	Firmicutes	Clostridia	Clostridiales	Lachnospiraceae	Shuttleworthia	0.0000%	0.0004%	0.3421	NA
Bacteria	Firmicutes	Clostridia	Clostridiales	Peptostreptococcaceae	Paraclostridium	0.0000%	0.0014%	0.3421	NA
Bacteria	Firmicutes	Clostridia	Clostridiales	Ruminococcaceae	Fournierella	0.0000%	0.0014%	0.3421	NA
Bacteria	Firmicutes	Negativicutes	Selenomonadales	Selenomonadaceae	Selenomonas	0.0000%	0.0015%	0.3421	NA
Bacteria	Firmicutes	Negativicutes	Veillonellales	Veillonellaceae	Caecibacter	0.0000%	0.0015%	0.3421	NA
Bacteria	Firmicutes	Tissierellia	Tissierellales	Tissierellaceae	Urmitella	0.0000%	0.0195%	0.3421	NA
Bacteria	Proteobacteria	Alphaproteobacteria	Rhodobacterales	Hyphomonadaceae	Fretibacter	0.0000%	0.0013%	0.3421	NA
Bacteria	Proteobacteria	Alphaproteobacteria	Rhodospirillales	Rhodospirillaceae	Aestuariispira	0.0000%	0.0011%	0.3421	NA
Bacteria	Proteobacteria	Gammaproteobacteria	Pseudomonadales	Moraxellaceae	Acinetobacter	0.0000%	0.0014%	0.3421	NA
Archaea	Euryarchaeota	Methanobacteria	Methanobacteriales	Methanobacteriaceae	Methanobrevibacter	0.1128%	0.0032%	0.3492	NA
Bacteria	Firmicutes	Erysipelotrichia	Erysipelotrichales	Erysipelotrichaceae	Massiliomicrobiota	0.0063%	0.0022%	0.3497	NA
Bacteria	Firmicutes	Clostridia	Clostridiales	Ruminococcaceae	Anaerotruncus	0.0022%	0.0021%	0.3497	NA
Bacteria	Firmicutes	Clostridia	Clostridiales	Defluviitaleaceae	Defluviitalea	0.0017%	0.0011%	0.3497	NA
Bacteria	Firmicutes	Clostridia	Clostridiales	Lachnospiraceae	Lachnoanaerobaculum	0.0011%	0.0014%	0.3497	NA
Bacteria	Proteobacteria	Betaproteobacteria	Neisseriales	Neisseriaceae	Neisseria	0.0010%	0.0007%	0.3497	NA
Bacteria	Firmicutes	Negativicutes	Veillonellales	Veillonellaceae	Megasphaera	0.0010%	0.0059%	0.3721	NA
Bacteria	Firmicutes	Bacilli	Lactobacillales	Streptococcaceae	Lactococcus	0.0702%	0.0211%	0.4162	NA
Bacteria	Firmicutes	Bacilli	Lactobacillales	Carnobacteriaceae	Granulicatella	0.0124%	0.0069%	0.4325	NA
Bacteria	Firmicutes	Clostridia	Clostridiales	Ruminococcaceae	Sporobacter	0.6028%	0.5215%	0.4389	NA
Bacteria	Firmicutes	Clostridia	Clostridiales	Lachnospiraceae	Anaerostipes	0.1351%	0.2504%	0.4395	NA
Bacteria	Firmicutes	Clostridia	Clostridiales	Ruminococcaceae	Flavonifractor	0.0290%	0.0127%	0.4420	NA
Bacteria	Firmicutes	Clostridia	Clostridiales	Ruminococcaceae	Paludicola	0.0320%	0.0056%	0.4450	NA
Bacteria	Firmicutes	Clostridia	Clostridiales	Clostridiales_Family_XIII._Incertae_Sedis	Ihubacter	0.0014%	0.0023%	0.4461	NA
Bacteria	Firmicutes	Clostridia	Clostridiales	Clostridiaceae	Lutispora	0.0182%	0.0075%	0.4635	NA
Bacteria	Proteobacteria	Betaproteobacteria	Burkholderiales	Sutterellaceae	Sutterella	0.1494%	0.2653%	0.4759	NA
Bacteria	Lentisphaerae	Lentisphaeria	Victivallales	Victivallaceae	Victivallis	0.0740%	0.0178%	0.4810	NA
Bacteria	Firmicutes	Clostridia	Clostridiales	Ruminococcaceae	Ethanoligenens	0.0529%	0.0113%	0.4835	NA

Bacteria	Firmicutes	Clostridia	Clostridiales	Clostridiaceae	Clostridium	0.5610%	0.3347%	0.4858	NA
Bacteria	Firmicutes	Clostridia	Clostridiales	Ruminococcaceae		0.6175%	1.5618%	0.4899	NA
Bacteria	Firmicutes	Erysipelotrichia	Erysipelotrichales	Erysipelotrichaceae	Turicibacter	0.0641%	0.0226%	0.5060	NA
Bacteria	Firmicutes	Clostridia	Clostridiales	Ruminococcaceae	Pseudoflavonifractor	0.0310%	0.0161%	0.5247	NA
Bacteria	Proteobacteria	Betaproteobacteria	Burkholderiales	Comamonadaceae	Comamonas	0.1258%	0.0060%	0.5341	NA
Bacteria	Firmicutes	Clostridia	Clostridiales	Hungateiclostridiaceae	Saccharofermentans	0.0081%	0.0013%	0.5341	NA
Bacteria	Firmicutes	Clostridia	Clostridiales	Clostridiales_Family_XIII._Incertae_Sedis	Anaerovorax	0.0003%	0.0023%	0.5341	NA
Bacteria	Firmicutes	Clostridia	Clostridiales	Ruminococcaceae	Negativibacillus	0.0062%	0.0034%	0.5356	NA
Bacteria	Firmicutes	Bacilli	Bacillales		Gemella	0.0017%	0.0011%	0.5356	NA
Bacteria	Actinobacteria	Actinobacteria	Micrococcales	Micrococcaceae	Rothia	0.0113%	0.0128%	0.5488	NA
Bacteria	Firmicutes	Erysipelotrichia	Erysipelotrichales	Erysipelotrichaceae	Coprobacillus	0.0093%	0.0314%	0.5614	NA
Bacteria	Firmicutes	Tissierellia	Tissierellales	Peptoniphilaceae	Anaerococcus	0.0049%	0.0004%	0.5737	NA
Bacteria	Firmicutes	Clostridia	Clostridiales	Catabacteriaceae	Catabacter	0.0027%	0.0008%	0.5737	NA
Bacteria	Firmicutes	Clostridia	Clostridiales	Clostridiaceae	Hungatella	0.0025%	0.0008%	0.5737	NA
Bacteria	Firmicutes	Negativicutes	Acidaminococcales	Acidaminococcaceae	Acidaminococcus	0.0018%	0.0075%	0.5737	NA
Bacteria	Actinobacteria	Actinobacteria	Actinomycetales	Actinomycetaceae	Schaalia	0.0275%	0.0013%	0.5917	NA
Bacteria	Proteobacteria	Betaproteobacteria	Burkholderiales	Sutterellaceae	Parasutterella	0.0070%	0.0342%	0.5917	NA
Bacteria	Firmicutes	Tissierellia	Tissierellales	Tissierellaceae	Anaerosalibacter	0.1748%	0.1944%	0.6147	NA
Bacteria	Firmicutes	Bacilli	Bacillales	Bacillaceae	Pseudogracilibacillus	0.1377%	3.3097%	0.6147	NA
Bacteria	Firmicutes	Bacilli	Bacillales	Bacillaceae	Virgibacillus	0.0335%	4.1551%	0.6147	NA
Bacteria	Bacteroidetes	Bacteroidia	Bacteroidales	Prevotellaceae	Massiliprevotella	0.0081%	0.0410%	0.6147	NA
Bacteria	Bacteroidetes	Bacteroidia	Bacteroidales	Porphyromonadaceae	Gabonibacter	0.0054%	0.0035%	0.6147	NA
Bacteria	Cyanobacteria		Oscillatoriales	Oscillatoriaceae	Aerosakkonema	0.0037%	0.0006%	0.6147	NA
Bacteria	Actinobacteria	Coriobacteriia	Coriobacteriales	Atopobiaceae	Atopobium	0.0020%	0.0009%	0.6147	NA
Bacteria	Firmicutes	Clostridia	Clostridiales	Peptostreptococcaceae	Intestinibacter	0.0015%	0.0014%	0.6147	NA
Bacteria	Firmicutes	Clostridia	Clostridiales	Lachnospiraceae	Eisenbergiella	0.0281%	0.0100%	0.6211	NA
Bacteria	Firmicutes	Clostridia	Clostridiales	Ruminococcaceae	Neglecta	0.0604%	0.0410%	0.6499	NA
Bacteria	Firmicutes	Bacilli	Bacillales	Bacillaceae	Bacillus	0.1177%	0.0239%	0.6547	NA
Bacteria	Firmicutes	Bacilli	Lactobacillales	Leuconostocaceae	Weissella	0.0226%	0.0503%	0.6689	NA
Bacteria	Bacteroidetes	Bacteroidia	Bacteroidales	Prevotellaceae	Prevotellamassilia	0.1176%	0.5085%	0.6705	NA
Bacteria	Actinobacteria	Coriobacteriia	Coriobacteriales	Coriobacteriaceae	Collinsella	0.1618%	0.2126%	0.7247	NA
Bacteria	Verrucomicrobia	Verrucomicrobiae	Verrucomicrobiales	Akkermansiaceae	Akkermansia	0.3907%	0.0579%	0.7508	NA
Bacteria	Proteobacteria	Betaproteobacteria	Burkholderiales	Oxalobacteraceae	Oxalobacter	0.0049%	0.0113%	0.7594	NA
Bacteria	Firmicutes	Negativicutes	Selenomonadales	Selenomonadaceae	Megamonas	0.2103%	0.2678%	0.7984	NA
Bacteria	Actinobacteria	Coriobacteriia	Eggerthellales	Eggerthellaceae	Raoultibacter	0.0045%	0.0156%	0.8970	NA
Bacteria	Verrucomicrobia	Verrucomicrobiae	Verrucomicrobiales	Verrucomicrobiaceae	Fucophilus	0.0505%	0.1250%	0.9150	NA
Bacteria	Firmicutes	Clostridia	Clostridiales		Howardella	0.0092%	0.0117%	0.9229	NA
Bacteria	Firmicutes	Negativicutes	Veillonellales	Veillonellaceae	Allisonella	0.0015%	0.0040%	0.9306	NA
Bacteria	Firmicutes	Clostridia	Clostridiales	Lachnospiraceae	Hespellia	0.0012%	0.0017%	0.9306	NA
Bacteria	Actinobacteria	Actinobacteria	Actinomycetales	Actinomycetaceae	Actinomyces	0.0008%	0.0009%	0.9379	NA
Bacteria	Firmicutes	Bacilli	Lactobacillales	Enterococcaceae	Enterococcus	0.0565%	0.1066%	0.9430	NA
Bacteria	Firmicutes	Clostridia	Clostridiales	Hungateiclostridiaceae	Ruminiclostridium	0.2257%	0.0277%	0.9433	NA
Bacteria	Firmicutes	Clostridia	Clostridiales		Flintibacter	0.4052%	0.5530%	0.9461	NA
Bacteria	Firmicutes	Clostridia	Clostridiales		Monoglobus	0.0134%	0.0050%	0.9653	NA
Bacteria	Proteobacteria	Gammaproteobacteria	Enterobacterales	Hafniaceae	Hafnia	0.0046%	0.0172%	0.9653	NA
Bacteria	Proteobacteria	Gammaproteobacteria	Enterobacterales	Enterobacteriaceae	Cronobacter	0.0017%	0.0023%	0.9793	NA
Bacteria	Bacteroidetes	Bacteroidia	Bacteroidales	Prevotellaceae	Alloprevotella	0.1297%	0.0761%	0.9846	NA
Bacteria	Firmicutes	Bacilli	Bacillales	Bacillaceae	Oceanobacillus	0.5282%	0.1395%	1.0000	NA

Bacteria	Firmicutes	Clostridia	Clostridiales	Lachnospiraceae	Butyrivibrio	0.3387%	0.1876%	1.0000	NA
Bacteria	Tenericutes	Mollicutes	Anaeroplasmatales	Anaeroplasmataceae	Anaeroplasma	0.2001%	0.0003%	1.0000	NA
Bacteria	Firmicutes	Bacilli	Bacillales	Planococcaceae	Rummeliibacillus	0.1441%	2.2250%	1.0000	NA
Bacteria	Firmicutes	Tissierellia	Tissierellales	Tissierellaceae	Tissierella	0.0227%	0.0058%	1.0000	NA
Bacteria	Bacteroidetes	Bacteroidia	Bacteroidales	Barnesiellaceae	Coprobacter	0.0131%	0.0047%	1.0000	NA
Bacteria	Tenericutes	Mollicutes	Entomoplasmatales	Spiroplasmataceae	Spiroplasma	0.0080%	0.0009%	1.0000	NA
Bacteria	Proteobacteria	Gammaproteobacteria	Aeromonadales	Succinivibrionaceae	Succinivibrio	0.0035%	0.0004%	1.0000	NA
Bacteria	Firmicutes	Clostridia	Clostridiales	Ruminococcaceae	Caproiciproducens	0.0029%	0.0018%	1.0000	NA
Bacteria	Synergistetes	Synergistia	Synergistales	Synergistaceae	Pyramidobacter	0.0012%	0.0057%	1.0000	NA
Bacteria	Actinobacteria	Actinobacteria	Bifidobacteriales	Bifidobacteriaceae	Scardovia	0.0009%	0.0003%	1.0000	NA
Bacteria	Actinobacteria	Coriobacteriia	Coriobacteriales	Coriobacteriaceae	Senegalimassilia	0.0009%	0.0003%	1.0000	NA
Bacteria	Actinobacteria	Coriobacteriia	Eggerthellales	Eggerthellaceae	Adlercreutzia	0.0007%	0.0015%	1.0000	NA
Bacteria	Firmicutes	Clostridia	Clostridiales	Clostridiales_Family_XIII._Incertae_Sedis	Mogibacterium	0.0007%	0.0004%	1.0000	NA
Bacteria	Firmicutes	Negativicutes	Selenomonadales	Selenomonadaceae	Mitsuokella	0.0005%	0.0021%	1.0000	NA

Supplementary Table 2. Full taxonomic comparative analysis of oral microbiome

Kingdom	Phylum	Class	Order	Family	Genus	STEMI	Control	Unadjusted P-value	FDR-adjusted P-value
Bacteria	Bacteroidetes	Bacteroidia	Bacteroidales	Bacteroidaceae	Bacteroides	0.8630%	0.0522%	0.0002	0.0065
Bacteria	Firmicutes	Bacilli	Lactobacillales	Carnobacteriaceae	Granulicatella	1.2894%	0.4991%	0.005	0.0745
Bacteria	Bacteroidetes	Bacteroidia	Bacteroidales	Prevotellaceae	Alloprevotella	0.5845%	0.4838%	0.0247	0.1957
Bacteria	Proteobacteria	Gammaproteobacteria	Pasteurellales	Pasteurellaceae	Haemophilus	5.7948%	12.4364%	0.0284	0.1957
Bacteria	Firmicutes	Bacilli	Lactobacillales	Streptococcaceae	Streptococcus	24.2930%	38.2492%	0.218	0.1957
Bacteria	Bacteroidetes	Flavobacteriia	Flavobacteriales	Flavobacteriaceae	Capnocytophaga	3.8586%	1.4046%	0.0418	0.2088
Bacteria	Bacteroidetes	Bacteroidia	Bacteroidales	Porphyromonadaceae	Porphyromonas	5.0350%	2.8145%	0.0675	0.2894
Bacteria	Actinobacteria	Actinobacteria	Actinomycetales	Actinomycetaceae	Schaalia	0.3601%	0.3117%	0.1162	0.4357
Bacteria	Proteobacteria	Epsilonproteobacteria	Campylobacterales	Campylobacteraceae	Campylobacter	0.9428%	0.4859%	0.149	0.4965
Bacteria	Synergistetes	Synergistia	Synergistales	Synergistaceae	Fretibacterium	0.3883%	0.1282%	0.1661	0.4982
Bacteria	Bacteroidetes	Bacteroidia	Bacteroidales	Tannerellaceae	Tannerella	0.4506%	0.4670%	0.2122	0.5688
Bacteria	Proteobacteria	Betaproteobacteria	Neisseriales	Neisseriaceae	Neisseria	13.0602%	8.3580%	0.2365	0.5688
Bacteria	Proteobacteria	Betaproteobacteria	Burkholderiales	Burkholderiaceae	Lautropia	8.0017%	2.0863%	0.2465	0.5688
Bacteria	Bacteroidetes	Bacteroidia	Bacteroidales	Prevotellaceae	Prevotella	7.3281%	5.3570%	0.3112	0.6222
Unassigned	Unassigned	Unassigned	Unassigned	Unassigned	Unassigned	0.9949%	1.0226%	0.3112	0.6222
Bacteria	Spirochaetes	Spirochaetia	Spirochaetales	Spirochaetaceae	Treponema	1.0122%	0.6108%	0.3319	0.6222
Bacteria	Fusobacteria	Fusobacteriia	Fusobacteriales	Fusobacteriaceae	Fusobacterium	2.0903%	2.1788%	0.3752	0.6434
Bacteria	Bacteroidetes	Bacteroidia	Bacteroidales	Prevotellaceae	Metaprevotella	0.3626%	0.1027%	0.386	0.6434
Bacteria	Firmicutes	Negativicutes	Selenomonadales	Selenomonadaceae	Selenomonas	0.3760%	0.0985%	0.4089	0.6456
Bacteria	Fusobacteria	Fusobacteriia	Fusobacteriales	Leptotrichiaceae	Leptotrichia	1.9613%	1.6069%	0.5411	0.8117
Bacteria	Firmicutes	Clostridia	Clostridiales	Peptostreptococcaceae	Filifactor	0.3377%	0.2165%	0.6002	0.8259
Bacteria	Firmicutes	Bacilli	Bacillales		Gemella	1.4470%	2.5724%	0.6056	0.8259
Bacteria	Firmicutes	Negativicutes	Veillonellales	Veillonellaceae	Veillonella	2.5755%	3.3427%	0.6447	0.8409
Bacteria	Actinobacteria	Actinobacteria	Actinomycetales	Actinomycetaceae	Actinomyces	0.4914%	0.9196%	0.687	0.8587
Bacteria	Tenericutes	Mollicutes	Mycoplasmatales	Mycoplasmataceae	Mycoplasma	0.5024%	0.3352%	0.7232	0.8678
Bacteria	Proteobacteria	Gammaproteobacteria	Cardiobacteriales	Cardiobacteriaceae	Cardiobacterium	0.3217%	0.2421%	0.7526	0.8684
Bacteria	Proteobacteria	Gammaproteobacteria	Pasteurellales	Pasteurellaceae	Aggregatibacter	0.6564%	0.4789%	0.8699	0.9604
Bacteria	Actinobacteria	Actinobacteria	Micrococcales	Micrococcaceae	Rothia	6.4780%	6.4948%	0.9107	0.9604
Bacteria	Firmicutes	Negativicutes	Selenomonadales	Selenomonadaceae	Centipeda	0.4612%	0.1010%	0.9284	0.9604
Bacteria	Firmicutes	Clostridia	Clostridiales	Lachnospiraceae	Oribacterium	0.3058%	0.2975%	0.9799	0.9799
Bacteria	Firmicutes	Clostridia	Clostridiales	Ruminococcaceae	Faecalibacterium	0.2419%	0.0003%	0.0013	NA
Bacteria	Firmicutes	Clostridia	Clostridiales	Lachnospiraceae	Lachnoclostridium	0.1371%	0.0034%	0.0015	NA
Bacteria	Proteobacteria	Betaproteobacteria	Burkholderiales	Comamonadaceae	Acidovorax	0.0473%	0.0011%	0.0018	NA
Bacteria	Fusobacteria	Fusobacteriia	Fusobacteriales	Fusobacteriaceae	Cetobacterium	0.0769%	0.0000%	0.007	NA
Bacteria	Bacteroidetes	Bacteroidia	Bacteroidales	Tannerellaceae	Parabacteroides	0.0119%	0.0000%	0.007	NA
Bacteria	Firmicutes	Clostridia	Clostridiales	Lachnospiraceae	Kineothrix	0.0348%	0.0000%	0.0138	NA
Bacteria	Firmicutes	Clostridia	Clostridiales	Ruminococcaceae	Ruminococcus	0.0163%	0.0000%	0.0138	NA
Bacteria	Firmicutes	Clostridia	Clostridiales	Lachnospiraceae	Johnsonella	0.0767%	0.0102%	0.0208	NA
Bacteria	Proteobacteria	Gammaproteobacteria	Enterobacterales	Enterobacteriaceae	Escherichia	0.0205%	0.0032%	0.0225	NA
Bacteria	Bacteroidetes	Flavobacteriia	Flavobacteriales	Flavobacteriaceae	Flavobacterium	0.1541%	0.0000%	0.0267	NA
Bacteria	Deinococcus.Thermus	Deinococci	Deinococcales	Deinococcaceae	Deinococcus	0.0412%	0.0000%	0.0267	NA
Bacteria	Proteobacteria	Deltaproteobacteria	Desulfuromonadales	Desulfuromonadaceae	Pelobacter	0.0169%	0.0000%	0.0267	NA
Bacteria	Firmicutes	Clostridia	Clostridiales	Lachnospiraceae		0.0243%	0.0006%	0.0301	NA
Bacteria	Firmicutes	Clostridia	Clostridiales	Oscillospiraceae	Oscillibacter	0.0482%	0.0005%	0.0351	NA

Bacteria	Proteobacteria	Gammaproteobacteria	Pseudomonadales	Moraxellaceae	Acinetobacter	0.0125%	0.0339%	0.0376	NA
Bacteria	Bacteroidetes	Bacteroidia	Bacteroidales	Rikenellaceae	Alistipes	0.2090%	0.0002%	0.0457	NA
Bacteria	Proteobacteria	Betaproteobacteria	Burkholderiales	Sutterellaceae	Sutterella	0.1147%	0.0002%	0.0457	NA
Bacteria	Firmicutes	Clostridia	Clostridiales	Lachnospiraceae	Anaerotaenia	0.0197%	0.0000%	0.0508	NA
Bacteria	Firmicutes	Clostridia	Clostridiales	Ruminococcaceae	Gemmiger	0.0165%	0.0000%	0.0508	NA
Bacteria	Firmicutes	Negativicutes	Selenomonadales	Selenomonadaceae	Megamonas	0.0134%	0.0000%	0.0508	NA
Bacteria	Firmicutes	Negativicutes	Acidaminococcales	Acidaminococcaceae	Phascolarctobacterium	0.0122%	0.0000%	0.0508	NA
Bacteria	Bacteroidetes	Bacteroidia	Bacteroidales	Prevotellaceae	Paraprevotella	0.0081%	0.0000%	0.0508	NA
Bacteria	Proteobacteria	Betaproteobacteria	Burkholderiales	Oxalobacteraceae	Massilia	0.0056%	0.0000%	0.0508	NA
Bacteria	Firmicutes	Erysipelotrichia	Erysipelotrichales	Erysipelotrichaceae	Holdemanella	0.0046%	0.0000%	0.0508	NA
Bacteria	Firmicutes	Clostridia	Clostridiales	Ruminococcaceae	Sporobacter	0.0036%	0.0000%	0.0508	NA
Bacteria	Firmicutes	Clostridia	Clostridiales	Lachnospiraceae	Coprococcus	0.0020%	0.0000%	0.0508	NA
Bacteria	Firmicutes	Erysipelotrichia	Erysipelotrichales	Erysipelotrichaceae	Bulleidia	0.0025%	0.0426%	0.0527	NA
Bacteria	Bacteroidetes	Bacteroidia	Bacteroidales	Barnesiellaceae	Barnesiella	0.0331%	0.0006%	0.0548	NA
Bacteria	Spirochaetes	Spirochaetia	Spirochaetales	Spirochaetaceae	Sphaerochaeta	0.0265%	0.0006%	0.0548	NA
Bacteria	Firmicutes	Bacilli	Lactobacillales	Lactobacillaceae	Lactobacillus	0.2343%	0.8772%	0.0583	NA
Bacteria	Proteobacteria	Gammaproteobacteria	Pseudomonadales	Pseudomonadaceae	Pseudomonas	0.0620%	0.0261%	0.0617	NA
Bacteria	Proteobacteria	Gammaproteobacteria	Pasteurellales	Pasteurellaceae	Mesocricetibacter	0.0000%	0.0020%	0.0668	NA
Bacteria	Bacteroidetes	Flavobacteriia	Flavobacteriales	Flavobacteriaceae	Cloacibacterium	0.1464%	0.0669%	0.0778	NA
Bacteria	Bacteroidetes	Bacteroidia	Bacteroidales	Prevotellaceae	Prevotellamassilia	0.0960%	0.0000%	0.0962	NA
Bacteria	Proteobacteria	Deltaproteobacteria	Myxococcales	Polyangiaceae	Chondromyces	0.0124%	0.0000%	0.0962	NA
Bacteria	Proteobacteria	Betaproteobacteria	Rhodocyclales	Rhodocyclaceae	Propionivibrio	0.0117%	0.0000%	0.0962	NA
Bacteria	Bacteroidetes	Bacteroidia	Bacteroidales	Odoribacteraceae	Butyricimonas	0.0079%	0.0000%	0.0962	NA
Bacteria	Firmicutes	Clostridia	Clostridiales	Lachnospiraceae	Anaerostipes	0.0071%	0.0000%	0.0962	NA
Bacteria	Firmicutes	Tissierellia	Tissierellales		Sporanaerobacter	0.0042%	0.0000%	0.0962	NA
Bacteria	Firmicutes	Clostridia	Clostridiales	Lachnospiraceae	Eisenbergiella	0.0030%	0.0000%	0.0962	NA
Bacteria	Firmicutes	Clostridia	Clostridiales	Lachnospiraceae	Fusicatenibacter	0.0019%	0.0000%	0.0962	NA
Bacteria	Firmicutes	Clostridia	Clostridiales		Flintibacter	0.0250%	0.0017%	0.0979	NA
Bacteria	Firmicutes	Clostridia	Clostridiales	Eubacteriaceae	Eubacterium	0.0130%	0.0008%	0.0979	NA
Bacteria	Proteobacteria	Alphaproteobacteria	Caulobacterales	Caulobacteraceae	Brevundimonas	0.0117%	0.0006%	0.0979	NA
Bacteria	Firmicutes	Bacilli	Lactobacillales	Streptococcaceae	Lactococcus	0.0047%	0.0185%	0.1	NA
Bacteria	Actinobacteria	Actinobacteria	Propionibacteriales	Propionibacteriaceae	Cutibacterium	0.0280%	0.0143%	0.1032	NA
Bacteria	Proteobacteria	Gammaproteobacteria	Pseudomonadales	Moraxellaceae	Moraxella	0.0293%	0.0060%	0.1084	NA
Bacteria	Firmicutes	Bacilli	Lactobacillales	Aerococcaceae	Abiotrophia	0.2484%	0.0121%	0.1179	NA
Bacteria	Actinobacteria	Actinobacteria	Micrococcales	Dermacoccaceae	Calidifontibacter	0.0000%	0.0023%	0.1423	NA
Bacteria	Actinobacteria	Coriobacteriia	Eggerthellales	Eggerthellaceae	Cryptobacterium	0.0000%	0.0004%	0.1423	NA
Bacteria	Proteobacteria	Gammaproteobacteria	Enterobacterales	Enterobacteriaceae	Citrobacter	0.0874%	0.0376%	0.1486	NA
Bacteria	Bacteroidetes	Flavobacteriia	Flavobacteriales	Flavobacteriaceae	Chryseobacterium	0.0765%	0.0430%	0.1523	NA
Bacteria	Bacteroidetes	Bacteroidia	Bacteroidales	Muribaculaceae	Muribaculum	0.0255%	0.0545%	0.1528	NA
Bacteria	Bacteroidetes	Bacteroidia	Bacteroidales	Odoribacteraceae	Odoribacter	0.0984%	0.0160%	0.1571	NA
Bacteria	Proteobacteria	Gammaproteobacteria	Cardiobacteriales	Cardiobacteriaceae	Suttonella	0.0137%	0.0002%	0.1721	NA
Bacteria	Verrucomicrobia	Verrucomicrobiae	Verrucomicrobiales	Akkermansiaceae	Akkermansia	0.0092%	0.0002%	0.1721	NA
Bacteria	Firmicutes	Clostridia	Clostridiales	Eubacteriaceae	Pseudoramibacter	0.0051%	0.0001%	0.1721	NA
Bacteria	Actinobacteria	Actinobacteria	Bifidobacteriales	Bifidobacteriaceae	Bifidobacterium	0.0356%	0.1707%	0.1816	NA
Bacteria	Proteobacteria	Betaproteobacteria	Burkholderiales	Comamonadaceae	Limnohabitans	0.0559%	0.0000%	0.1833	NA
Bacteria	Actinobacteria	Actinobacteria	Micrococcales	Microbacteriaceae	Candidatus_Planktoluna	0.0527%	0.0000%	0.1833	NA
Bacteria	Firmicutes	Clostridia	Clostridiales	Clostridiaceae	Clostridium	0.0257%	0.0000%	0.1833	NA
Bacteria	Proteobacteria	Gammaproteobacteria	Cellvibrionales	Cellvibrionaceae	Cellvibrio	0.0217%	0.0000%	0.1833	NA
Bacteria	Actinobacteria	Coriobacteriia	Coriobacteriales	Coriobacteriaceae	Collinsella	0.0112%	0.0000%	0.1833	NA

Bacteria	Firmicutes	Bacilli	Lactobacillales	Lactobacillaceae	Pediococcus	0.0112%	0.0000%	0.1833	NA
Bacteria	Firmicutes	Clostridia	Clostridiales	Lachnospiraceae	Roseburia	0.0065%	0.0000%	0.1833	NA
Bacteria	Firmicutes	Clostridia	Clostridiales	Lachnospiraceae	Dorea	0.0038%	0.0000%	0.1833	NA
Bacteria	Firmicutes	Tissierellia	Tissierellales	Peptoniphilaceae	Anaerococcus	0.0038%	0.0000%	0.1833	NA
Bacteria	Proteobacteria	Gammaproteobacteria	Xanthomonadales	Xanthomonadaceae	Stenotrophomonas	0.0028%	0.0000%	0.1833	NA
Bacteria	Proteobacteria	Alphaproteobacteria	Rhodospirillales	Acetobacteraceae	Acetobacter	0.0026%	0.0000%	0.1833	NA
Bacteria	Actinobacteria	Actinobacteria	Micrococcales	Micrococcaceae	Glutamicibacter	0.0022%	0.0000%	0.1833	NA
Bacteria	Firmicutes	Clostridia	Clostridiales	Clostridiaceae	Butyricicoccus	0.0020%	0.0000%	0.1833	NA
Bacteria	Proteobacteria	Gammaproteobacteria	Alteromonadales	Shewanellaceae	Shewanella	0.0020%	0.0000%	0.1833	NA
Bacteria	Firmicutes	Clostridia	Clostridiales	Clostridiaceae	Hungatella	0.0015%	0.0000%	0.1833	NA
Bacteria	Proteobacteria	Betaproteobacteria	Burkholderiales	Comamonadaceae	Kinneretia	0.0012%	0.0000%	0.1833	NA
Bacteria	Firmicutes	Clostridia	Clostridiales	Lachnospiraceae	Anaerotignum	0.0009%	0.0000%	0.1833	NA
Bacteria	Proteobacteria	Gammaproteobacteria	Enterobacterales	Morganellaceae	Providencia	0.0007%	0.0000%	0.1833	NA
Bacteria	Firmicutes	Clostridia	Clostridiales	Clostridiales_Family_XIII._Incertae_Sedis	Ihubacter	0.0358%	0.0029%	0.2001	NA
Bacteria	Fusobacteria	Fusobacteriia	Fusobacteriales	Leptotrichiaceae	Sneathia	0.1468%	0.0763%	0.2182	NA
Bacteria	Firmicutes	Bacilli	Lactobacillales	Enterococcaceae	Enterococcus	0.0003%	0.0014%	0.2354	NA
Bacteria	Firmicutes	Clostridia	Clostridiales	Defluviitaleaceae	Defluviitalea	0.1262%	0.0708%	0.2409	NA
Bacteria	Proteobacteria	Deltaproteobacteria	Desulfobacterales	Desulfobulbaceae	Desulfobulbus	0.1157%	0.0103%	0.251	NA
Bacteria	Actinobacteria	Actinobacteria	Bifidobacteriales	Bifidobacteriaceae	Scardovia	0.0007%	0.0733%	0.2703	NA
Bacteria	Bacteroidetes	Bacteroidia	Bacteroidales	Paludibacteraceae	Paludibacter	0.1433%	0.1264%	0.2812	NA
Bacteria	Firmicutes	Erysipelotrichia	Erysipelotrichales	Erysipelotrichaceae	Solobacterium	0.0185%	0.0170%	0.2835	NA
Bacteria	Firmicutes	Tissierellia	Tissierellales	Peptoniphilaceae	Peptoniphilus	0.0030%	0.0046%	0.2991	NA
Bacteria	Firmicutes	Clostridia	Clostridiales	Lachnospiraceae	Shuttleworthia	0.0099%	0.0082%	0.305	NA
Bacteria	Actinobacteria	Actinobacteria	Corynebacteriales	Dietziaceae	Dietzia	0.0000%	0.0005%	0.3168	NA
Bacteria	Actinobacteria	Actinobacteria	Micrococcales	Micrococcaceae	Nesterenkonia	0.0000%	0.0011%	0.3168	NA
Bacteria	Actinobacteria	Actinobacteria	Propionibacteriales	Nocardioidaceae	Aeromicrobium	0.0000%	0.0007%	0.3168	NA
Bacteria	Actinobacteria	Actinobacteria	Propionibacteriales	Nocardioidaceae	Micropruina	0.0000%	0.0006%	0.3168	NA
Bacteria	Bacteroidetes	Flavobacteriia	Flavobacteriales	Flavobacteriaceae	Myroides	0.0000%	0.0003%	0.3168	NA
Bacteria	Cyanobacteria		Synechococcales	Chamaesiphonaceae	Chamaesiphon	0.0000%	0.0005%	0.3168	NA
Bacteria	Firmicutes	Bacilli	Bacillales	Bacillaceae	Virgibacillus	0.0000%	0.0002%	0.3168	NA
Bacteria	Firmicutes	Clostridia	Clostridiales	Gracilibacteraceae	Gracilibacter	0.0000%	0.0002%	0.3168	NA
Bacteria	Firmicutes	Clostridia	Clostridiales	Lachnospiraceae	Abyssivirga	0.0000%	0.0008%	0.3168	NA
Bacteria	Firmicutes	Erysipelotrichia	Erysipelotrichales	Erysipelotrichaceae	Faecalibaculum	0.0000%	0.0025%	0.3168	NA
Bacteria	Proteobacteria	Alphaproteobacteria	Rhizobiales	Hyphomicrobiaceae	Pelagibacterium	0.0000%	0.0007%	0.3168	NA
Bacteria	Proteobacteria	Alphaproteobacteria	Rhizobiales	Xanthobacteraceae	Azorhizobium	0.0000%	0.0069%	0.3168	NA
Bacteria	Proteobacteria	Alphaproteobacteria	Rhodospirillales	Acetobacteraceae	Roseomonas	0.0000%	0.0013%	0.3168	NA
Bacteria	Proteobacteria	Betaproteobacteria	Burkholderiales	Burkholderiaceae	Cupriavidus	0.0000%	0.0042%	0.3168	NA
Bacteria	Proteobacteria	Gammaproteobacteria	Pasteurellales	Pasteurellaceae	Actinobacillus	0.0000%	0.0011%	0.3168	NA
Bacteria	Proteobacteria	Gammaproteobacteria	Xanthomonadales	Xanthomonadaceae	Luteimonas	0.0000%	0.0005%	0.3168	NA
Bacteria	Proteobacteria	Gammaproteobacteria	Xanthomonadales	Xanthomonadaceae	Pseudoxanthomonas	0.0000%	0.0085%	0.3168	NA
Bacteria	Proteobacteria	Betaproteobacteria	Neisseriales	Neisseriaceae	Kingella	0.1824%	0.2455%	0.3244	NA
Bacteria	Firmicutes	Clostridia	Clostridiales	Peptostreptococcaceae	Peptostreptococcus	0.2068%	0.0800%	0.325	NA
Bacteria	Proteobacteria	Betaproteobacteria	Neisseriales	Neisseriaceae	Eikenella	0.1104%	0.1075%	0.3316	NA
Bacteria	Firmicutes	Clostridia	Clostridiales	Lachnospiraceae	Lachnoanaerobaculum	0.2618%	0.3593%	0.3322	NA
Bacteria	Proteobacteria	Deltaproteobacteria	Desulfovibrionales	Desulfomicrobiaceae	Desulfomicrobium	0.0071%	0.0024%	0.3346	NA
Bacteria	Actinobacteria	Actinobacteria	Propionibacteriales	Propionibacteriaceae	Pseudopropionibacterium	0.0033%	0.0103%	0.3434	NA
Bacteria	Tenericutes	Mollicutes	Acholeplasmatales	Acholeplasmataceae	Acholeplasma	0.0082%	0.0009%	0.3475	NA
Bacteria	Proteobacteria	Betaproteobacteria	Neisseriales	Neisseriaceae	Snodgrassella	0.0153%	0.0360%	0.3582	NA
Bacteria	Firmicutes	Erysipelotrichia	Erysipelotrichales	Erysipelotrichaceae	Turicibacter	0.1406%	0.0000%	0.365	NA

Bacteria	Proteobacteria	Gammaproteobacteria	Aeromonadales	Aeromonadaceae	Aeromonas	0.0180%	0.0000%	0.365	NA
Bacteria	Proteobacteria	Alphaproteobacteria	Rhodospirillales	Rhodospirillaceae	Dongia	0.0177%	0.0000%	0.365	NA
Bacteria	Cyanobacteria		Nostocales	Rivulariaceae	Calothrix	0.0175%	0.0000%	0.365	NA
Bacteria	Bacteroidetes	Cytophagia	Cytophagales	Cytophagaceae	Emticicia	0.0162%	0.0000%	0.365	NA
Bacteria	Proteobacteria	Alphaproteobacteria	Rhizobiales	Hyphomicrobiaceae	Devosia	0.0144%	0.0000%	0.365	NA
Bacteria	Bacteroidetes	Flavobacteriia	Flavobacteriales	Crocinitomicaceae	Fluviicola	0.0131%	0.0000%	0.365	NA
Bacteria	Proteobacteria	Betaproteobacteria	Burkholderiales	Oxalobacteraceae	Undibacterium	0.0128%	0.0000%	0.365	NA
Bacteria	Proteobacteria	Betaproteobacteria	Burkholderiales	Burkholderiaceae	Polynucleobacter	0.0127%	0.0000%	0.365	NA
Bacteria	Proteobacteria	Betaproteobacteria	Burkholderiales	Comamonadaceae	Simplicispira	0.0097%	0.0000%	0.365	NA
Bacteria	Proteobacteria	Betaproteobacteria	Burkholderiales	Burkholderiaceae	Hydromonas	0.0095%	0.0000%	0.365	NA
Bacteria	Proteobacteria	Betaproteobacteria	Burkholderiales	Burkholderiaceae	Limnobacter	0.0090%	0.0000%	0.365	NA
Bacteria	Proteobacteria	Betaproteobacteria	Rhodocyclales	Azonexaceae	Dechloromonas	0.0090%	0.0000%	0.365	NA
Bacteria	Proteobacteria	Epsilonproteobacteria	Campylobacterales	Helicobacteraceae	Wolinella	0.0061%	0.0000%	0.365	NA
Bacteria	Actinobacteria	Actinobacteria	Corynebacteriales	Mycobacteriaceae	Mycolicibacterium	0.0047%	0.0000%	0.365	NA
Bacteria	Proteobacteria	Betaproteobacteria	Rhodocyclales	Azonexaceae	Quatrionicoccus	0.0047%	0.0000%	0.365	NA
Bacteria	Acidobacteria	Solibacteres	Solibacterales	Bryobacteraceae	Paludibaculum	0.0042%	0.0000%	0.365	NA
Bacteria	Actinobacteria	Actinobacteria	Micrococcales	Microbacteriaceae	Rhodoluna	0.0040%	0.0000%	0.365	NA
Bacteria	Proteobacteria	Oligoflexia	Bdellovibrionales	Bdellovibrionaceae	Bdellovibrio	0.0038%	0.0000%	0.365	NA
Bacteria	Proteobacteria	Gammaproteobacteria	Thiotrichales	Piscirickettsiaceae	Methylophaga	0.0036%	0.0000%	0.365	NA
Bacteria	Bacteroidetes	Flavobacteriia	Flavobacteriales	Flavobacteriaceae	Mesoflavibacter	0.0035%	0.0000%	0.365	NA
Bacteria	Bacteroidetes	Flavobacteriia	Flavobacteriales	Flavobacteriaceae	Polaribacter	0.0035%	0.0000%	0.365	NA
Bacteria	Proteobacteria	Epsilonproteobacteria	Campylobacterales	Campylobacteraceae	Arcobacter	0.0035%	0.0000%	0.365	NA
Bacteria	Firmicutes	Clostridia	Clostridiales	Lachnospiraceae	Tyzzerella	0.0034%	0.0000%	0.365	NA
Bacteria	Bacteroidetes	Bacteroidia	Bacteroidales	Bacteroidaceae	Mediterranea	0.0033%	0.0000%	0.365	NA
Bacteria	Proteobacteria	Gammaproteobacteria	Enterobacterales	Morganellaceae	Morganella	0.0030%	0.0000%	0.365	NA
Bacteria	Firmicutes	Clostridia	Clostridiales		Fenollaria	0.0030%	0.0000%	0.365	NA
Bacteria	Firmicutes	Clostridia	Clostridiales	Hungateiclostridiaceae	Hungateiclostridium	0.0030%	0.0000%	0.365	NA
Bacteria	Firmicutes	Clostridia	Clostridiales	Lachnospiraceae	Lachnospira	0.0029%	0.0000%	0.365	NA
Bacteria	Proteobacteria	Alphaproteobacteria	Rhizobiales	Methylobacteriaceae	Methylorubrum	0.0029%	0.0000%	0.365	NA
Bacteria	Proteobacteria	Betaproteobacteria	Burkholderiales	Alcaligenaceae	Alcaligenes	0.0027%	0.0000%	0.365	NA
Bacteria	Bacteroidetes	Bacteroidia	Bacteroidales	Barnesiellaceae	Coprobacter	0.0023%	0.0000%	0.365	NA
Bacteria	Chloroflexi	Caldilineae	Caldilineales	Caldilineaceae	Litorilinea	0.0022%	0.0000%	0.365	NA
Bacteria	Proteobacteria	Betaproteobacteria	Burkholderiales	Sutterellaceae	Duodenibacillus	0.0021%	0.0000%	0.365	NA
Bacteria	Bacteroidetes	Saprospiria	Saprospirales	Haliscomenobacteraceae	Phaeodactylibacter	0.0018%	0.0000%	0.365	NA
Bacteria	Proteobacteria	Gammaproteobacteria	Enterobacterales	Morganellaceae	Proteus	0.0017%	0.0000%	0.365	NA
Bacteria	Actinobacteria	Actinobacteria	Micrococcales	Dermabacteraceae	Brachybacterium	0.0016%	0.0000%	0.365	NA
Bacteria	Proteobacteria	Betaproteobacteria	Burkholderiales	Comamonadaceae	Rhodoferax	0.0016%	0.0000%	0.365	NA
Bacteria	Proteobacteria	Alphaproteobacteria	Sphingomonadales	Sphingomonadaceae	Sphingorhabdus	0.0014%	0.0000%	0.365	NA
Bacteria	Firmicutes	Clostridia	Clostridiales	Ruminococcaceae	Ethanoligenens	0.0013%	0.0000%	0.365	NA
Bacteria	Proteobacteria	Alphaproteobacteria	Rhodospirillales	Rhodospirillaceae	Aliidongia	0.0013%	0.0000%	0.365	NA
Bacteria	Firmicutes	Bacilli	Bacillales	Bacillaceae	Polygonibacillus	0.0013%	0.0000%	0.365	NA
Bacteria	Proteobacteria	Alphaproteobacteria	Sphingomonadales	Sphingomonadaceae	Sphingobium	0.0012%	0.0000%	0.365	NA
Bacteria	Bacteroidetes	Chitinophagia	Chitinophagales	Chitinophagaceae	Nemorincola	0.0012%	0.0000%	0.365	NA
Bacteria	Firmicutes	Clostridia	Clostridiales	Lachnospiraceae	Herbinix	0.0011%	0.0000%	0.365	NA
Bacteria	Proteobacteria	Gammaproteobacteria	Alteromonadales	Colwelliaceae	Colwellia	0.0011%	0.0000%	0.365	NA
Bacteria	Firmicutes	Clostridia	Clostridiales		Intestinimonas	0.0010%	0.0000%	0.365	NA
Bacteria	Proteobacteria	Betaproteobacteria	Burkholderiales	Comamonadaceae	Xenophilus	0.0009%	0.0000%	0.365	NA
Bacteria	Proteobacteria	Alphaproteobacteria	Sphingomonadales	Sphingomonadaceae	Sphingopyxis	0.0009%	0.0000%	0.365	NA
Bacteria	Firmicutes	Clostridia	Clostridiales	Defluviitaleaceae	Vallitalea	0.0008%	0.0000%	0.365	NA

Bacteria	Firmicutes	Clostridia	Halanaerobiales	Halanaerobiaceae	Halocella	0.0008%	0.0000%	0.365	NA
Bacteria	Chlamydiae	Chlamydiia	Parachlamydiales	Criblamydiaceae	Estrella	0.0008%	0.0000%	0.365	NA
Bacteria	Firmicutes	Bacilli	Bacillales	Alicyclobacillaceae	Alicyclobacillus	0.0008%	0.0000%	0.365	NA
Bacteria	Firmicutes	Bacilli	Bacillales	Paenibacillaceae	Thermobacillus	0.0008%	0.0000%	0.365	NA
Bacteria	Firmicutes	Bacilli	Bacillales	Planococcaceae	Sporosarcina	0.0008%	0.0000%	0.365	NA
Bacteria	Firmicutes	Bacilli	Bacillales	Paenibacillaceae	Cohnella	0.0007%	0.0000%	0.365	NA
Bacteria	Proteobacteria	Alphaproteobacteria	Rhodobacterales	Rhodobacteraceae	Rubellimicrobium	0.0006%	0.0000%	0.365	NA
Bacteria	Proteobacteria	Alphaproteobacteria	Rhodobacterales	Rhodobacteraceae	Rhodobacter	0.0006%	0.0000%	0.365	NA
Bacteria	Proteobacteria	Alphaproteobacteria	Rhizobiales	Rhizobiaceae	Shinella	0.0006%	0.0000%	0.365	NA
Bacteria	Candidatus_Melainabacteria		Vampirovibrionales		Vampirovibrio	0.0006%	0.0000%	0.365	NA
Bacteria	Proteobacteria	Betaproteobacteria	Neisseriales	Neisseriaceae	Conchiformibius	0.0005%	0.0000%	0.365	NA
Bacteria	Firmicutes	Erysipelotrichia	Erysipelotrichales	Erysipelotrichaceae	Catenibacterium	0.0005%	0.0000%	0.365	NA
Bacteria	Bacteroidetes	Bacteroidia	Bacteroidales	Dysgonamonadaceae	Proteiniphilum	0.0004%	0.0000%	0.365	NA
Bacteria	Armatimonadetes	Fimbriimonadia	Fimbriimonadales	Fimbriimonadaceae	Fimbriimonas	0.0004%	0.0000%	0.365	NA
Archaea	Euryarchaeota	Thermoplasmata	Methanomassiliicoccales	Methanomassiliicoccaceae	Methanomassiliicoccus	0.0004%	0.0000%	0.365	NA
Bacteria	Proteobacteria	Gammaproteobacteria	Pasteurellales	Pasteurellaceae	Frederiksenia	0.0004%	0.0000%	0.365	NA
Bacteria	Actinobacteria	Actinobacteria	Geodermatophilales	Geodermatophilaceae	Blastococcus	0.0004%	0.0000%	0.365	NA
Bacteria	Firmicutes	Erysipelotrichia	Erysipelotrichales	Erysipelotrichaceae	Eggerthia	0.0004%	0.0000%	0.365	NA
Bacteria	Proteobacteria	Gammaproteobacteria	Aeromonadales	Succinivibrionaceae	Succinivibrio	0.0003%	0.0000%	0.365	NA
Bacteria	Actinobacteria	Actinobacteria	Frankiales	Motilibacteraceae	Motilibacter	0.0002%	0.0000%	0.365	NA
Bacteria	Proteobacteria	Deltaproteobacteria	Desulfuromonadales	Geobacteraceae	Geothermobacter	0.0002%	0.0000%	0.365	NA
Bacteria	Firmicutes	Clostridia	Clostridiales	Ruminococcaceae	Neglecta	0.0002%	0.0000%	0.365	NA
Bacteria	Firmicutes	Bacilli	Bacillales	Bacillaceae	Pseudogracilibacillus	0.0002%	0.0000%	0.365	NA
Bacteria	Firmicutes	Clostridia	Clostridiales	Lachnospiraceae	Faecalicatena	0.0001%	0.0000%	0.365	NA
Bacteria	Firmicutes	Clostridia	Clostridiales	Lachnospiraceae	Pseudobutyrivibrio	0.0001%	0.0000%	0.365	NA
Bacteria	Firmicutes	Clostridia	Clostridiales	Ruminococcaceae	Flavonifractor	0.0001%	0.0000%	0.365	NA
Bacteria	Actinobacteria	Coriobacteriia	Eggerthellales	Eggerthellaceae	Eggerthella	0.0001%	0.0000%	0.365	NA
Bacteria	Proteobacteria	Deltaproteobacteria	Desulfovibrionales	Desulfovibrionaceae	Desulfovibrio	0.0109%	0.0186%	0.3709	NA
Bacteria	Firmicutes	Clostridia	Clostridiales	Peptostreptococcaceae	Peptoanaerobacter	0.0455%	0.0314%	0.3719	NA
Bacteria	Actinobacteria	Actinobacteria	Actinomycetales	Actinomycetaceae	Mobiluncus	0.0009%	0.0004%	0.3735	NA
Bacteria	Firmicutes	Tissierellia	Tissierellales	Peptoniphilaceae	Parvimonas	0.1261%	0.1455%	0.3985	NA
Bacteria	Bacteroidetes	Flavobacteriia	Flavobacteriales	Flavobacteriaceae	Bergeyella	0.0014%	0.0010%	0.4006	NA
Bacteria	Firmicutes	Clostridia	Clostridiales	Clostridiales_Family_XIII._Incertae_Sedis		0.1874%	0.1232%	0.4127	NA
Bacteria	Firmicutes	Bacilli	Lactobacillales	Leuconostocaceae	Leuconostoc	0.0107%	0.0004%	0.4197	NA
Bacteria	Firmicutes	Clostridia	Clostridiales	Peptococcaceae	Peptococcus	0.0058%	0.0007%	0.4624	NA
Bacteria	Proteobacteria	Alphaproteobacteria	Rhodobacterales	Rhodobacteraceae	Paracoccus	0.0327%	0.0020%	0.4685	NA
Bacteria	Firmicutes	Clostridia	Clostridiales	Clostridiales_Family_XIII._Incertae_Sedis	Mogibacterium	0.0018%	0.0070%	0.47	NA
Bacteria	Proteobacteria	Alphaproteobacteria	Sphingomonadales	Sphingomonadaceae	Sphingomonas	0.0004%	0.0095%	0.4808	NA
Bacteria	Actinobacteria	Actinobacteria	Corynebacteriales	Gordoniaceae	Gordonia	0.0004%	0.0016%	0.4808	NA
Bacteria	Proteobacteria	Gammaproteobacteria	Xanthomonadales	Xanthomonadaceae	Xanthomonas	0.0001%	0.0044%	0.4808	NA
Bacteria	Bacteroidetes	Bacteroidia	Bacteroidales		Phocaeicola	0.0364%	0.0108%	0.5276	NA
Bacteria	Firmicutes	Tissierellia	Tissierellales	Tissierellaceae		0.0222%	0.0053%	0.5328	NA
Bacteria	Actinobacteria	Actinobacteria	Corynebacteriales		Lawsonella	0.0209%	0.0060%	0.5328	NA
Bacteria	Synergistetes	Synergistia	Synergistales	Synergistaceae	Pyramidobacter	0.0071%	0.0029%	0.5537	NA
Bacteria	Firmicutes	Clostridia	Clostridiales	Hungateiclostridiaceae	Anaerobacterium	0.0718%	0.0607%	0.5775	NA
Bacteria	Firmicutes	Bacilli	Bacillales		Exiguobacterium	0.0279%	0.0009%	0.5921	NA
Bacteria	Actinobacteria	Actinobacteria	Micrococcales	Micrococcaceae	Micrococcus	0.0063%	0.0005%	0.5921	NA
Bacteria	Actinobacteria	Coriobacteriia	Eggerthellales	Eggerthellaceae	Slackia	0.0026%	0.0003%	0.5921	NA
Bacteria	Firmicutes	Erysipelotrichia	Erysipelotrichales	Erysipelotrichaceae	Erysipelatoclostridium	0.0015%	0.0002%	0.5921	NA

Bacteria	Firmicutes	Clostridia	Clostridiales	Lachnospiraceae	Catonella	0.2131%	0.0863%	0.5926	NA
Bacteria	Actinobacteria	Actinobacteria	Corynebacteriales	Nocardiaceae	Rhodococcus	0.0044%	0.0044%	0.6005	NA
Bacteria	Bacteroidetes	Bacteroidia	Bacteroidales	Bacteroidaceae	Anaerorhabdus	0.0009%	0.0021%	0.6005	NA
Bacteria	Actinobacteria	Coriobacteriia	Coriobacteriales	Atopobiaceae	Atopobium	0.1034%	0.2159%	0.6168	NA
Bacteria	Chloroflexi	Anaerolineae	Anaerolineales	Anaerolineaceae	Flexilinea	0.0012%	0.0006%	0.6317	NA
Bacteria	Proteobacteria	Betaproteobacteria	Burkholderiales	Comamonadaceae	Brachymonas	0.0726%	0.0330%	0.6436	NA
Bacteria	Firmicutes	Clostridia	Clostridiales	Hungateiclostridiaceae	Ruminiclostridium	0.0645%	0.0794%	0.6556	NA
Bacteria	Firmicutes	Clostridia	Clostridiales	Clostridiales_Family_XIII._Incertae_Sedis	Emergencia	0.0012%	0.0016%	0.6723	NA
Bacteria	Firmicutes	Negativicutes	Veillonellales	Veillonellaceae	Anaeroglobus	0.0299%	0.0922%	0.6811	NA
Bacteria	Firmicutes	Clostridia	Clostridiales	Clostridiaceae	Keratinibaculum	0.0383%	0.0010%	0.7035	NA
Bacteria	Firmicutes	Clostridia	Clostridiales	Ruminococcaceae		0.0038%	0.0010%	0.7035	NA
Bacteria	Firmicutes	Clostridia	Clostridiales	Clostridiales_Family_XIII._Incertae_Sedis	Aminipila	0.0405%	0.0323%	0.7062	NA
Bacteria	Actinobacteria	Actinobacteria	Corynebacteriales	Corynebacteriaceae	Corynebacterium	0.1962%	0.9926%	0.7149	NA
Bacteria	Firmicutes	Clostridia	Clostridiales	Christensenellaceae	Christensenella	0.0425%	0.0212%	0.7237	NA
Bacteria	Cyanobacteria		Oscillatoriales	Oscillatoriaceae	Aerosakkonema	0.1981%	0.3371%	0.743	NA
Bacteria	Firmicutes	Bacilli	Bacillales	Bacillaceae	Bacillus	0.0887%	0.0703%	0.7577	NA
Bacteria	Firmicutes	Negativicutes	Selenomonadales	Selenomonadaceae	Schwartzia	0.0105%	0.0027%	0.771	NA
Bacteria	Fusobacteria	Fusobacteriia	Fusobacteriales	Leptotrichiaceae	Streptobacillus	0.0707%	0.1706%	0.8	NA
Bacteria	Firmicutes	Negativicutes	Veillonellales	Veillonellaceae	Dialister	0.1725%	0.1370%	0.8005	NA
Bacteria	Firmicutes	Bacilli	Bacillales	Staphylococcaceae	Staphylococcus	0.2000%	0.0332%	0.8467	NA
Bacteria	Firmicutes	Clostridia	Clostridiales	Lachnospiraceae	Moryella	0.0281%	0.0189%	0.8611	NA
Bacteria	Firmicutes	Bacilli	Lactobacillales	Leuconostocaceae	Weissella	0.0251%	0.0021%	0.8685	NA
Bacteria	Actinobacteria	Coriobacteriia	Coriobacteriales	Atopobiaceae	Olsenella	0.0143%	0.0077%	0.8977	NA
Bacteria	Proteobacteria	Alphaproteobacteria	Sphingomonadales	Sphingomonadaceae	Novosphingobium	0.0155%	0.0292%	0.9456	NA
Bacteria	Actinobacteria	Actinobacteria	Bifidobacteriales	Bifidobacteriaceae	Parascardovia	0.0023%	0.0111%	0.9456	NA
Bacteria	Cyanobacteria		Nostocales	Tolypothrichaceae	Kryptousia	0.0001%	0.0748%	0.9456	NA
Bacteria	Actinobacteria	Actinobacteria	Actinomycetales	Actinomycetaceae	Actinotignum	0.0084%	0.2284%	0.9521	NA
Bacteria	Firmicutes	Clostridia	Clostridiales	Lachnospiraceae	Stomatobaculum	0.1229%	0.2220%	0.9597	NA
Bacteria	Firmicutes	Clostridia	Clostridiales	Lachnospiraceae	Butyrivibrio	0.0157%	0.0144%	0.9632	NA
Bacteria	Actinobacteria	Actinobacteria	Actinomycetales	Actinomycetaceae	Actinobaculum	0.0398%	0.0500%	0.9896	NA
Bacteria	Firmicutes	Negativicutes	Veillonellales	Veillonellaceae	Megasphaera	0.1544%	0.1219%	0.9897	NA
Bacteria	Proteobacteria	Alphaproteobacteria	Sphingomonadales	Sphingomonadaceae	Zymomonas	0.0207%	0.0007%	1	NA
Bacteria	Firmicutes	Clostridia	Clostridiales	Lachnospiraceae	Blautia	0.0078%	0.0016%	1	NA
Bacteria	Elusimicrobia	Endomicrobia	Endomicrobiales	Endomicrobiaceae	Endomicrobium	0.0022%	0.0005%	1	NA
Bacteria	Firmicutes	Clostridia	Clostridiales	Ruminococcaceae	Acetanaerobacterium	0.0016%	0.0001%	1	NA
Bacteria	Firmicutes	Clostridia	Clostridiales	Ruminococcaceae	Acetivibrio	0.0011%	0.0005%	1	NA
Bacteria	Proteobacteria	Alphaproteobacteria	Caulobacterales	Caulobacteraceae	Caulobacter	0.0009%	0.0007%	1	NA
Bacteria	Actinobacteria	Actinobacteria	Corynebacteriales	Nocardiaceae	Nocardia	0.0009%	0.0007%	1	NA
Bacteria	Proteobacteria	Betaproteobacteria	Burkholderiales	Comamonadaceae	Comamonas	0.0002%	0.0002%	1	NA
